# New insights into the structural role of EMILINs within the human skin microenvironment

**DOI:** 10.1038/s41598-024-81509-5

**Published:** 2024-12-05

**Authors:** Alvise Schiavinato, Fady Marcous, Alexandra V. Zuk, Douglas R. Keene, Sara F. Tufa, Laura M. Mosquera, Paola Zigrino, Cornelia Mauch, Beate Eckes, Katrien Francois, Julie De Backer, Nicolas Hunzelmann, Pia Moinzadeh, Thomas Krieg, Bert Callewaert, Gerhard Sengle

**Affiliations:** 1 Department of Pediatrics and Adolescent Medicine, Faculty of Medicine and University Hospital Cologne, University of Cologne, Joseph-Stelzmann-Str. 52, 50931 Cologne, Germany; 2 Center for Biochemistry, Faculty of Medicine and University Hospital Cologne, University of Cologne, 50931 Cologne, Germany; 3 Center for Molecular Medicine Cologne (CMMC), University of Cologne, 50931 Cologne, Germany; 4 Micro-Imaging Center, Shriners Children’s, Portland, OR 97239 USA; 5 Center for Medical Genetics, Ghent University Hospital, 9000 Ghent, Belgium; 6 Department of Pediatrics, Division of Pediatric Cardiology, Ghent University Hospital, 9000 Ghent, Belgium; 7 Department of Internal Medicine and Pediatrics, Ghent University Hospital, 9000 Ghent, Belgium; 8 Department of Dermatology, Faculty of Medicine and University Hospital Cologne, University of Cologne, 50931 Cologne, Germany; 9 Translational Matrix Biology, Faculty of Medicine, University Hospital Cologne, 50931 Cologne, Germany; 10 Department of Cardiovascular Surgery, Ghent University Hospital, 9000 Ghent, Belgium; 11 Department of Cardiology, Ghent University Hospital, 9000 Ghent, Belgium; 12 Cologne Excellence Cluster On Cellular Stress Responses in Ageing-Associated Diseases (CECAD), University of Cologne, 50931 Cologne, Germany; 13 Department of Biomolecular Medicine, Ghent University, 9000 Ghent, Belgium; 14 Cologne Center for Musculoskeletal Biomechanics (CCMB), 50931 Cologne, Germany

**Keywords:** Human skin, Dermal matrix, Elastic fibers, Oxytalan fibers, Basement membrane, Extracellular matrix proteins, UV exposure, Skin fibrosis, Connective tissue disease, Medical research, Molecular medicine, Skin diseases, Glycobiology, Glycobiology, Cells

## Abstract

Supramolecular extracellular matrix (ECM) networks play an essential role in skin architecture and function. Elastin microfibril interface-located proteins (EMILINs) comprise a family of three extracellular glycoproteins that serve as essential structural components of the elastin/fibrillin microfibril network, and exert crucial functions in cellular signaling. Little is known about the structural nature of EMILIN networks in skin. We therefore investigated the spatiotemporal localization of EMILIN-1, -2, -3 in human skin induced by aging, UV-exposure, fibrosis, and connective tissue disorder. Confocal immunofluorescence and immunogold electron microscopy analysis identified all EMILINs as components of elastic fibers and elastin-free oxytalan fibers inserted into the basement membrane (BM). Further, our ultrastructural analysis demonstrates cellular contacts of dermally localized EMILIN-1 positive fibers across the BM with the surface of basal keratinocytes. Analysis of skin biopsies and fibroblast cultures from fibrillin-1 deficient Marfan patients revealed that EMILINs require intact fibrillin-1 as deposition scaffold. In patients with scleroderma and the bleomycin-induced murine fibrosis model EMILIN-2 was upregulated. EMILIN-3 localizes to the tips of candelabra-like oxytalan fibers, and to specialized BMs engulfing hair follicles and sebaceous glands. Our data identify EMILINs as important markers to monitor rearrangements of the dermal ECM architecture induced by aging and pathological conditions.

## Introduction

EMILINs (Elastin Microfibrils Interface Located proteINs) constitute a family of three structurally homologous extracellular glycoproteins which endow microfibrillar microenvironments with unique functions. EMILINs were not only shown to regulate key cellular events, such as cell adhesion, migration, and proliferation^1–3^, but also to serve as unique modulators of extracellular signaling pathways^4^. EMILINs were reported to modulate proTGF-β processing^5,6^, activate the extrinsic apoptotic pathway^7,8^, and regulate Hedgehog and Wnt bioavailability^9,10^.

Initially, EMILIN-1 was discovered as a 115 kDa glycoprotein extractable from chicken aorta using harsh conditions such as 6 M guanidine HCl containing dithioerythritol^11^. Subsequently, two other extracellular proteins with high sequence similarity to EMILIN-1 were identified and therefore named EMILIN-2 and EMILIN-3^12,13^. Most structural information on EMILIN localization comes from ultrastructural studies in chick tissues, including skin, performed using specific antibodies against EMILIN-1^11^. These studies showed that EMILIN-1 is a component of elastic fibers mainly found in regions where elastin and fibrillin microfibrils (FMF) are in close contact^11^.

FMF are small diameter (10–12 nm) architectural elements with a characteristic “beads-on-a-string”-like appearance that are ubiquitously found in the connective tissue space^14,15^. FMF decorate the surface of elastic fibers but they also form independent networks of stretchable bundles, making them indispensable for conferring tensile strength and elasticity to tissues. The main building blocks of FMF are fibrillins (fibrillin-1 and -2), extracellular 350 kDa cysteine-rich glycoproteins with a conserved multidomain structure. FMF and elastic fibres are organized into tissue-specific architectures that reflect the mechanical demands of individual organ systems they direct. In normal dermis, elastic fibres are suspended in a three-dimensional basket-like lattice of FMF intersecting basement membranes at the dermal–epidermal junction and thus conferring pliability to the skin. Fibrillin has also been identified to be present within the lamina densa of the basement membrane, but there it is not assembled into FMF and adopts an unknown assembly form^16^. FMF have long been known to play a crucial role in directing elastogenesis; however, more recently FMF were shown to target and control the bioavailability of growth factors of the TGF-β superfamily, such as TGF-β1–3 and bone morphogenetic proteins (BMPs)^17–21^.

Most insight into the functional role of EMILINs was gained by the generation of murine knockout alleles. EMILIN-1 null mice display mild defects of elastic fibers^22^, increased blood pressure^6,23^, increased epidermal cell proliferation^1^, and defects of lymphatic vessels^24^. EMILIN-2 function has been mainly investigated in the context of tumor growth and neo-angiogenesis^8,25^. Another EMILIN-2 knockout mouse was generated and displays defects in platelets function and clot formation^26^. Morpholino-mediated knockdown of EMILIN-3 suggested a function of this protein during notochord development in zebrafish^9^. EMILIN-3 knockout mice have been established, however, analysis of their skin revealed no obvious phenotype^27^. While ablation of EMILIN-3 and EMILIN-2 did not result in any reported skin phenotypes, loss of EMILIN-1 in murine skin leads to dermal/ epidermal hyperproliferation and accelerated wound closure due to loss of α4β1 and α9β1 integrin binding resulting in activation of the phosphoinositide 3-kinase/AKT and ERK-1 and -2 signaling pathways^1^. Despite their unique functions, the exact role of EMILINs’ in the organization of the extracellular matrix (ECM) and therefore their structural and functional contribution to specialized extracellular supramolecular networks in the skin remains unknown. This information is critical for a better understanding of how skin microenvironments composed of intricate microfibrillar ECM networks control cell fate via cell-ECM communication.

We previously demonstrated that EMILINs are targeted to FMF in murine skin and that fibrillin-1 is required for the proper ECM deposition of both EMILIN-1 and -2^27,28^. This indicates that EMILIN-1 and -2 alterations may play a role in the pathomechanisms of the fibrillinopathies, where microfibril destabilization due to fibrillin-1 or -2 deficiency leads to multisystemic features characterized by reduced tissue integrity and global activation of growth factor signaling^29^. Recently, the first disease causing variant in the *EMILIN1* gene was reported in a patient with similar features to Marfan syndrome (MFS) such as aortic aneurysms, skeletal abnormalities and increased skin elasticity that is triggered by fibrillin-1 deficiency^30^. Moreover, we were able to show that familial pathogenic variants in *EMILIN1* cause *cutis laxa* and bone fragility by affecting the deposition of fibulin-4 and the activity of lysyl oxidase (LOX), a vital enzyme required for the stable formation of elastic fibers and the collagen network^31,32^. This observed clinical overlap of human EMILIN-1 and fibrillin-1 deficiency underpins our previous findings that EMILIN-1 is targeted to supramolecular networks composed of FMF and elastic fibers to which it confers functionality.

Here, by employing electron and confocal microscopy we gained further insight into the structural contribution of EMILINs to the ECM architecture in the papillary dermis at the dermal–epidermal junction of human skin. Further, we investigated how damage to the FMF/ elastic fiber system induced by sun exposure, aging, or pathological conditions such as fibrillin-1 deficiency in MFS, or scleroderma affect the distribution of EMILINs in human skin.

## Results

### Analysis of single-cell RNA sequencing data identifies EMILIN expression in distinct skin cell clusters

**Fig. 1:**
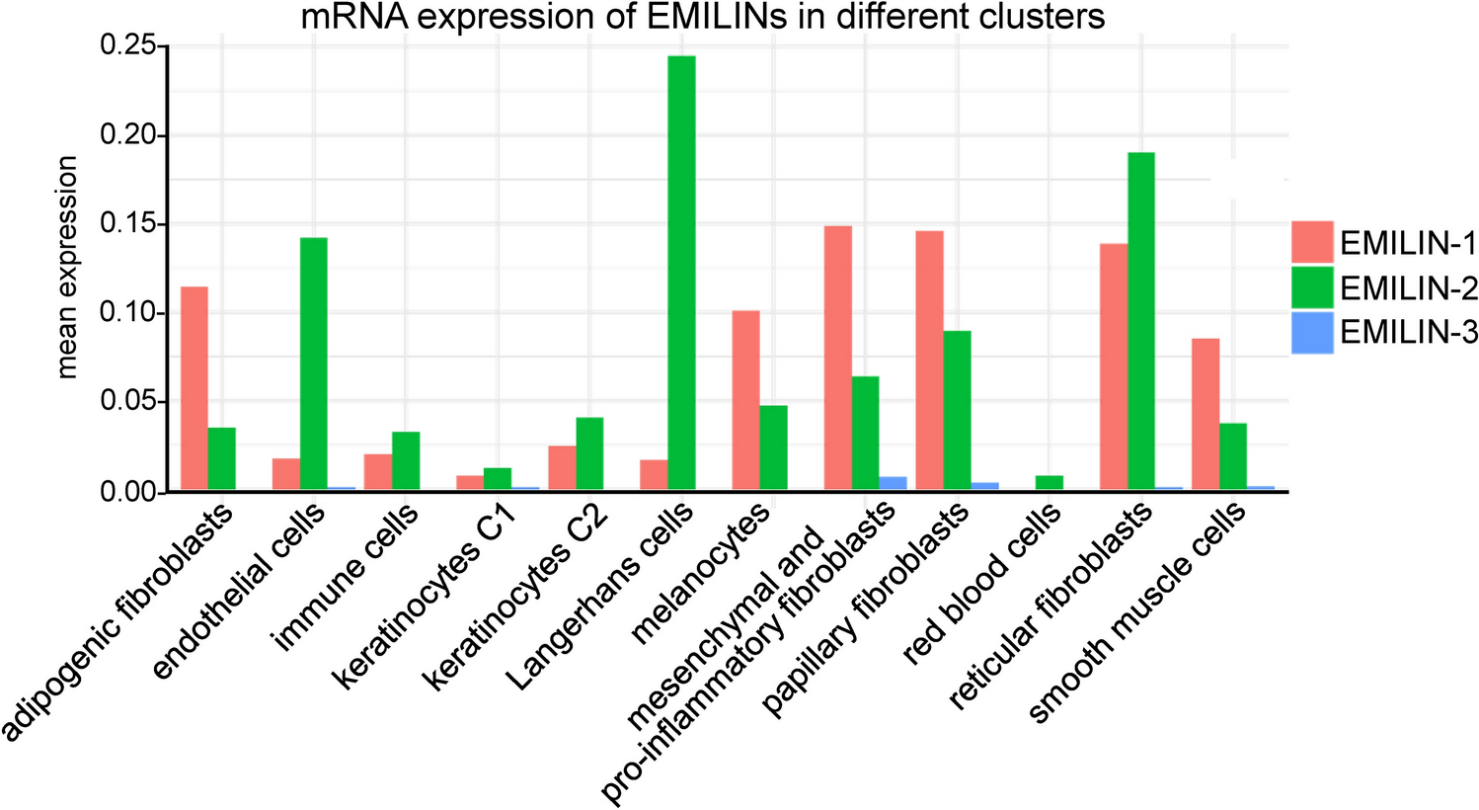
Single-cell RNA sequencing analysis reveals expression of EMILIN transcripts in distinct skin cell clusters. Mean expression levels of EMILIN-1, -2, and -3 transcripts (shown in rose, green, and light blue, respectively) across 12 identified skin cell clusters in the analyzed dataset^33^. Cell clusters were characterized based on the expression of specific marker genes (Supplementary Figs. S3 and S4, Supplementary Table S1). Mean expression levels were calculated as the average number of reads per cell for each *EMILIN* gene across clusters.

The cell type-specific expression of EMILINs in human skin remains poorly understood. To address this, we analyzed mRNA expression of EMILINs in different cellular clusters of the skin using a publicly available dataset from sun-protected areas of two “young” (25 and 27 years) and three “old” (53, 69, and 70 years) male Caucasian donors^33^. After applying quality control filters (Supplementary Fig. S1, see “Materials and methods” for details), we generated a uniform manifold approximation and projection (UMAP) plot that revealed 12 distinct cell clusters (Supplementary Fig. S2), corresponding to known cell types in human skin. Cluster identities were assigned by comparing established markers with the most representative genes expressed in each cluster (Supplementary Fig. S3, Supplementary Table S1). Our analysis revealed that EMILIN-1 and EMILIN-2 transcripts are predominantly expressed in various fibroblast subsets, including adipogenic, mesenchymal, pro-inflammatory, papillary, and reticular fibroblasts (Fig. 1). Notably, EMILIN-1 transcript levels are generally more abundant than EMILIN-2 across all fibroblast clusters, with the exception of reticular fibroblasts, where EMILIN-2 mRNA levels exceed those of EMILIN-1. Both genes were also expressed in smooth muscle cells, consistent with previous studies, and notably in melanocytes, a novel finding (Fig. 1). Interestingly, EMILIN-2 transcripts showed a distinct expression profile compared to EMILIN-1, with higher expression in endothelial and Langerhans cells. In contrast, immune cells and keratinocytes exhibited similar but low mRNA levels of both EMILIN-1 and EMILIN-2. EMILIN-3 was expressed at much lower levels overall, with the highest expression in mesenchymal, pro-inflammatory, and papillary fibroblasts (Fig. 1). Low transcript levels of EMILIN-3 were also detected in endothelial cells, keratinocytes, reticular fibroblasts, and smooth muscle cells.

Age-related differences in EMILIN expression were compared between young and old donors across the identified cell type clusters (Supplementary Figs. S4, S5) In aged skin, EMILIN-1 expression decreased by over 50% in adipogenic, mesenchymal, pro-inflammatory, and reticular fibroblasts but increased more than two-fold in papillary fibroblasts (Supplementary Fig. S5). Similarly, EMILIN-2 expression declined by 25–50% across most fibroblast subsets, with the exception of a smaller (~ 25%) reduction in mesenchymal, pro-inflammatory, papillary, and reticular fibroblasts (Supplementary Fig. S5). In contrast, EMILIN-3 expression was barely detectable in keratinocytes and mesenchymal fibroblasts of aged skin and absent in reticular fibroblasts and smooth muscle cells. However, it was significantly upregulated in papillary fibroblasts of aged skin (Supplementary Fig. S5).

### EMILINs localize to specific microenvironments in human skin

**Fig. 2:**
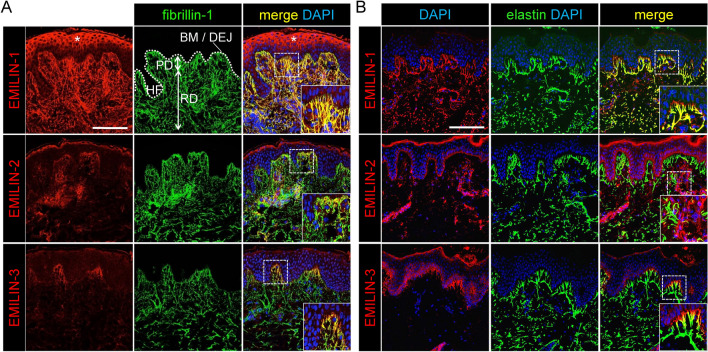
EMILINs have a specific localization and distribution in human skin as shown by confocal immunofluorescence and electron microscopy. EMILIN-1 and EMILIN-3, but not EMILIN-2, co-localize with fibrillin-1 and elastin. EMILIN-1 is distributed throughout the entire dermis, whereas EMILIN-3 is predominantly located at the tips of oxytalan fibers in close proximity to the basement membrane. (**A**) Co-localization of EMILINs with fibrillin-1 in dermis (parieto-occipital region, male donor, 15 years). Scale bar: 250 μm. Asterisks indicate nonspecific epidermal staining observed with the anti-EMILIN-1 antibody. (**B**) Co-localization of EMILINs with elastin (gluteal region, male donor, 29 years). Scale bar: 75 μm. Dotted white line indicates location of BM and DEJ. *BM* basement membrane, *DEJ* dermal epidermal junction, *HF* hair follicle, *PD* papillary dermis, *RD* reticular dermis.

**Fig. 3:**
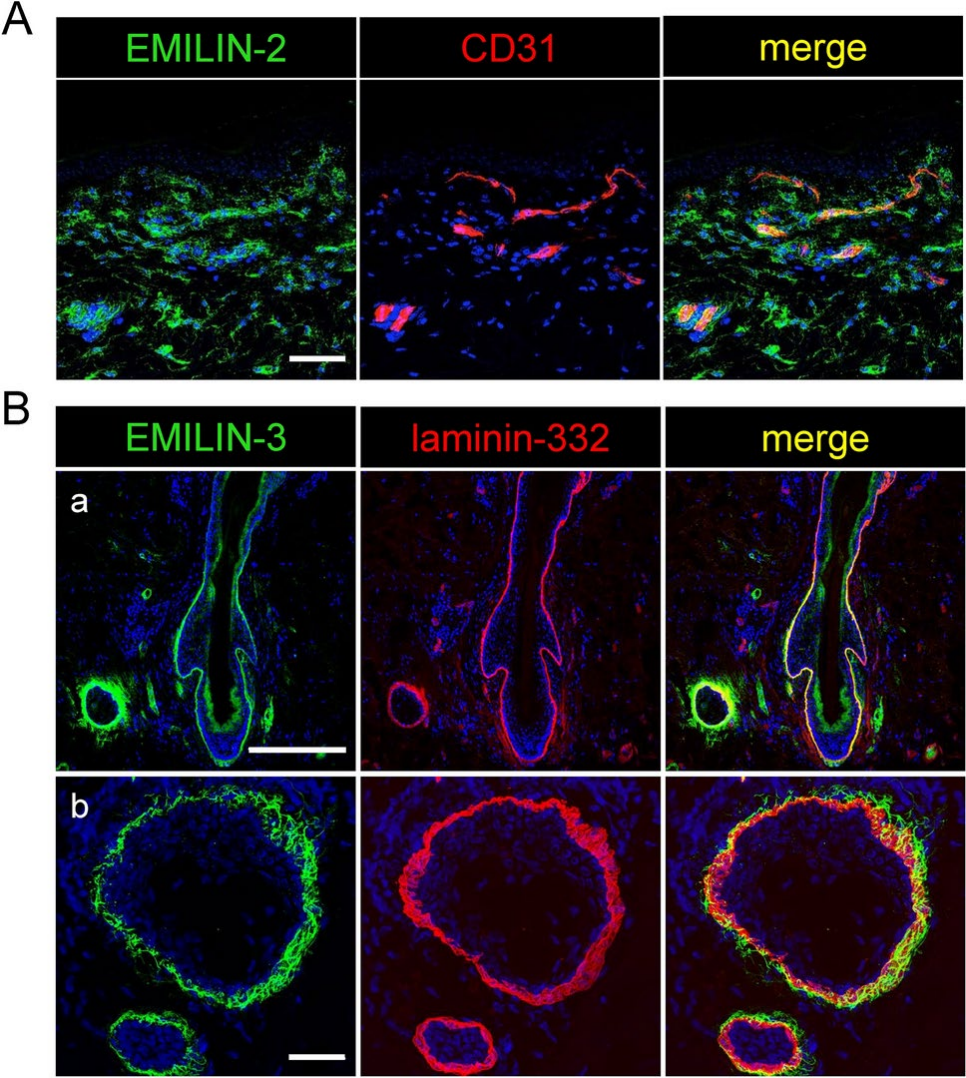
Localization of EMILIN-2 and -3 to specialized microenvironments of the skin. EMILIN-2 shows a perivascular localization in human dermis while EMILIN-3 was detected in skin appendages. (**A**) EMILIN-2 was co-stained with the endothelial cell marker CD31 and detected by confocal immunofluorescence microscopy on skin sections from the anterior axillary line of a 78-year old male donor. Scale bar: 75 μm. (**B**) EMILIN-3 and laminin-332 were co-stained on human skin sections and signals for both proteins were visualized by confocal immunofluorescence microscopy. Detected signals for EMILIN-3 localize to laminin-332 positive basement membranes surrounding (a) hair follicles and (b) sebaceous glands. Scale bars: 250 μm and 35 μm.

We next examined the localization and distribution of EMILIN proteins in human skin using specific antibodies. Co-immunolocalization studies of EMILINs with fibrillin-1 employing confocal immunofluorescence microscopy showed co-localization in sun-protected human dermis (Fig. 2A). In the reticular dermis, EMILIN-1 fibers run parallel to the epidermis, while in the papillary dermis they form candelabra-like structures perpendicularly oriented to the dermal epidermal junction (DEJ). Within these structures we found that EMILIN-1 co-localized with elastin (Fig. 2B). EMILIN-2 showed a more scattered distribution, with fibers irregularly interspersed throughout the dermis but no co-localization with elastin (Fig. 2B). However, closer inspection by electron microscopy (EM) in 19-month-old skin after immunogold labeling identified solely low intensity label of elastin associated FMF (Fig. S6). Co-immunostaining with the endothelial marker CD31 demonstrated a perivascular localization of EMILIN-2 in the dermal microvasculature of 78-year-old human skin (Fig. 3). This finding is supported by the presence of EMILIN-2 RNA transcripts in endothelial cells, where the other two EMILINs showed negligible expression (Fig. 1).

**Fig. 4:**
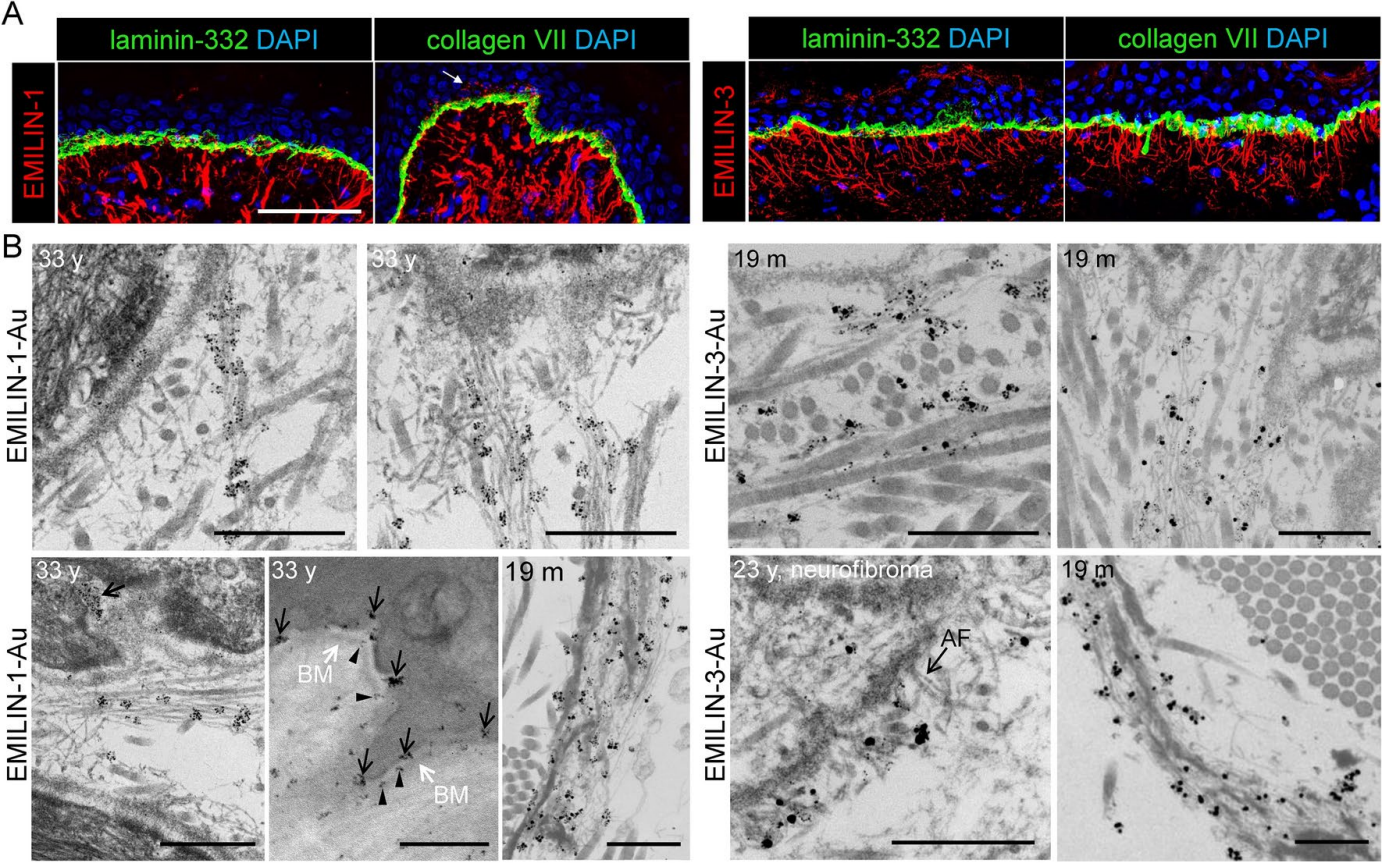
Specific localization of EMILIN-1 and -3 at the DEJ. EMILIN-1 and EMILIN-3 appear to localize within the BM. (**A**) Confocal immunofluorescence microscopy showing co-localization of EMILIN-1 and EMILIN-3 with laminin-332 and collagen VII at the DEJ (anterior axillary line, male donor, 78 years). White arrows point to EMILIN-1 signals beyond the BM. Scale bar: 75 μm. (**B**) Immunogold localization of EMILIN-1 and -3 in 33 year- (cheek), 19 month- (digit), and 23 year-old neurofibroma skin showing BM duplication. Scale bar: 500 nm. AF: anchoring fibrils, BM: basement membrane.

Consistent with RNA expression data, EMILIN-3 exhibited a restricted localization in the papillary dermis, predominantly co-localizing with thin fibrillin-1-positive oxytalan fibers lacking elastin Fig. 2B). These fibers form the uppermost portions of the candelabra-like structures that insert perpendicularly into the basement membrane (BM) (Fig. 2B). EMILIN-3 fibers were also abundantly present in specialized ECM microenvironments around hair follicles and sebaceous glands in close contact with BMs (Fig. 3). Further, co-immunofluorescence analysis revealed that EMILIN-1 and -3 both showed partial co-localization with laminin-332 and collagen VII suggesting their presence within the BM to provide anchorage of oxytalan fibers (Fig. 4A). To further investigate the exact distribution of EMILINs within the DEJ at higher resolution we employed immune-gold electron microscopy. Gold-labeled EMILIN-1 and -3 antibodies decorated long bundles of FMF intersecting into the BM (Fig. 4B and Supplemental Video S1). In proximity to elastin, EMILIN-1 localization was at the elastin core, while EMILIN-3 was solely detected at elastin associated FMF (Fig. 4B). Interestingly, immunogold EM also showed EMILIN-1 deposits beyond the BM on the basal surface of basal keratinocytes (Fig. 4B). Upon BM remodeling, as observed in a neurofibroma skin biopsy (Fig. S2), we found EMILIN-3 localization with anchoring fibrils (Fig. 4B, Fig. S7).

### Altered distribution of EMILINs due to aging and sun exposure

**Fig. 5:**
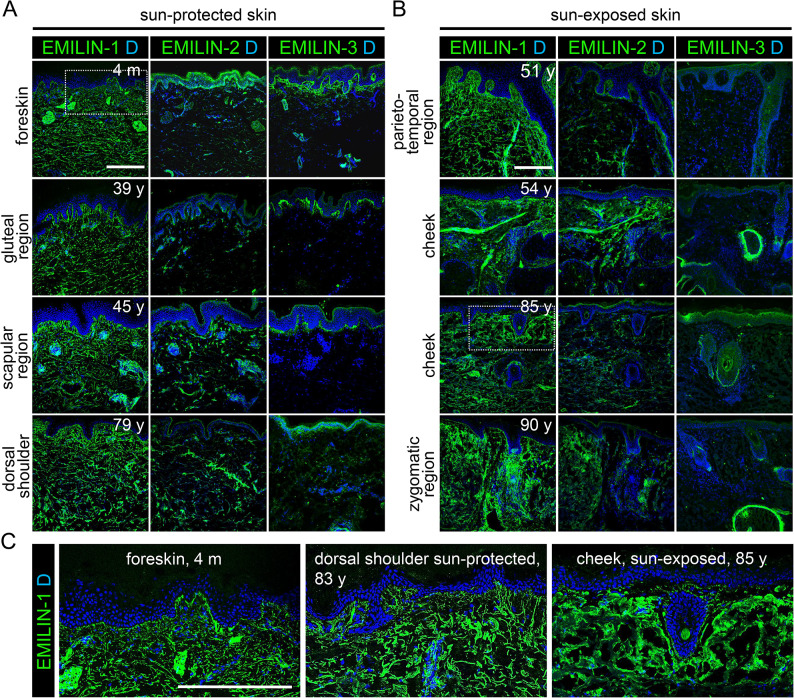
Altered localization of EMILINs in aged and sun-exposed skin. Ageing and photoageing lead to fragmentation of EMILIN-1 and EMILIN-3 fibers. (**A**) Localization of EMILINs in skin from donors of increasing age. (**B**) Localization of EMILINs in sun-exposed skin. Donor age and localization of skin biopsy are indicated. (**C**) (left) Localization of EMILIN-1 at the DEJ. Elongated EMILIN-1 fibers inserting from the dermis into the BM are not yet prominent at 4 month of age. (middle) In aged but sun-protected skin EMILIN-1 positive fibers appear to be often fragmented and show especially at the position of oxytalan fibers a punctate pattern. (right) In aged and sun-exposed skin elongated EMILIN-1 positive fibers are absent from the DEJ. Overall EMILIN-1 distribution appears to be more diffuse without a clear indication of linear deposition into discrete fibers. Images shown in left and right represent three-fold magnifications of the areas marked by white dashed boxes shown in (**A**) and (**B**). DAPI (**D**) marks nuclei. Ages of donors and anatomical localizations of taken biopsies are indicated. Scale bars: 250 μm.

Previously, it was reported that oxytalan fibers undergo degeneration in aged and photoaged skin due to the action of elastolytic enzymes^15,34^. Interestingly, we observed a small increase in EMILIN-2 staining intensity in the dermis with increased age (Fig. 5A). In sun-exposed skin, EMILIN-1 and EMILIN-2 showed a relative increase in deposition with reduced presence of fibrillar structures particularly in the papillary dermis, while EMILIN-3, unlike in sun-protected skin, was absent from the DEJ (Fig. 5B). At older age, EMILIN-3 fibers appeared to be shorter and fewer with decreased staining intensity (Fig. 5A). Closer inspection of EMILIN-1 signals at the DEJ revealed that elongated EMILIN-1 fibers inserting from the dermis into the BM are not yet prominent at 4 month of age (Fig. 5C). In aged but sun-protected skin, EMILIN-1 positive fibers appeared to be often fragmented and showed especially at the position of oxytalan fibers a punctate pattern (Fig. 5C). In aged and sun-exposed skin, the absence of elongated EMILIN-1 positive fibers from the DEJ was observed, alongside a more diffuse distribution of EMILIN-1 without distinct linear deposition into discrete fibers (Fig. 5C).

### Fibrillin-1 deficiency affects integrity of EMILIN-1, -2, -3 networks in skin of MFS patients

**Fig. 6:**
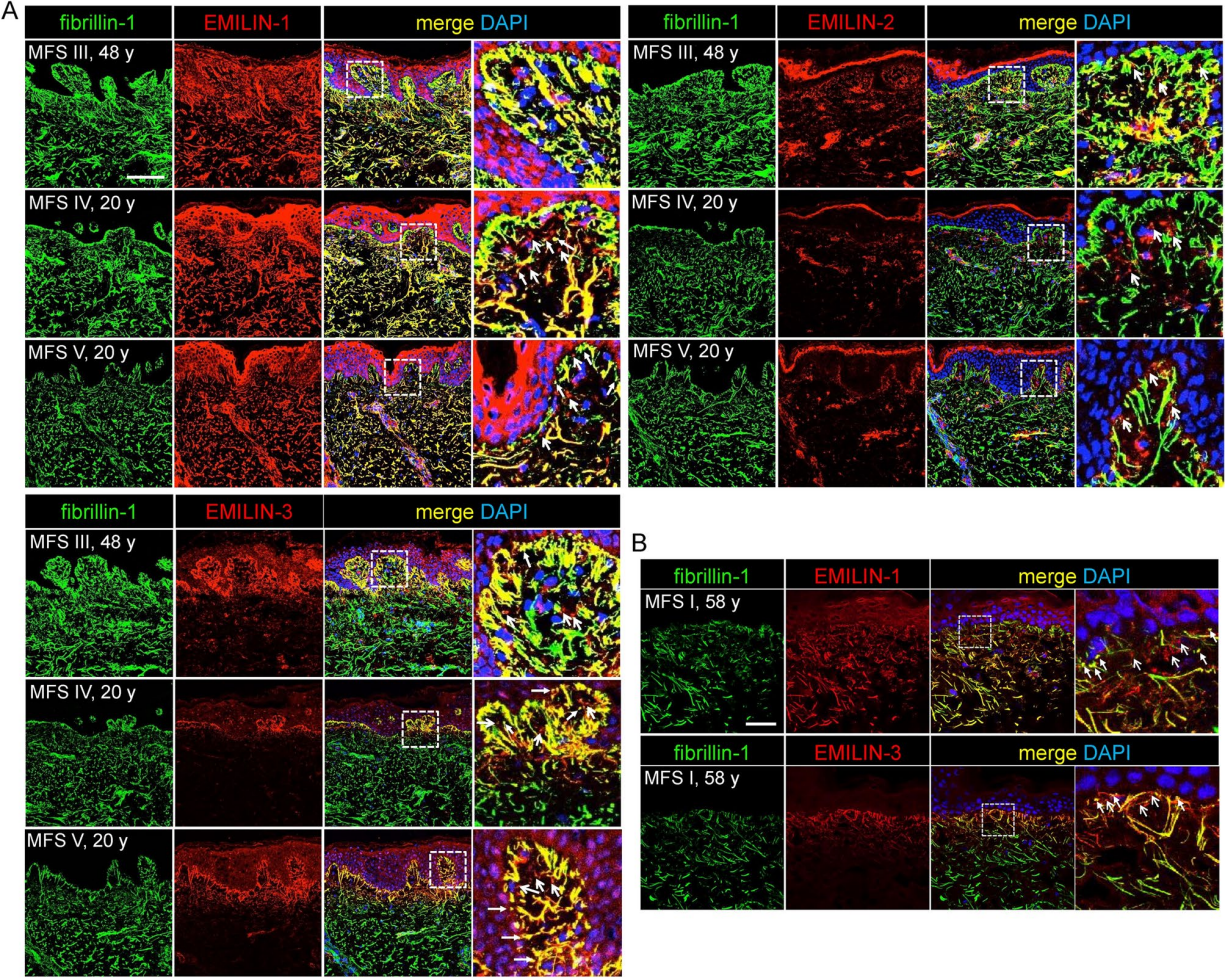
The presence of mutant fibrillin-1 affects the integrity of EMILIN networks in skin of patients with MFS. Fibrillin-1 deficiency negatively impacts the structural integrity of EMILIN-1, -2, and -3 fiber networks. EMILINs were detected together with fibrillin-1 in the dermis of three MFS patients (I-III) by confocal immunofluorescence microscopy. The appearance of non-colocalizing dotted signals suggests that degradation of fibrillin fibers due to fbrillin-1 deficiency leads to dissociation of EMILINs from this network. (**A**) Colocalization studies of EMILIN-1, -2, and -3 with fibrillin-1 showed impaired EMILIN network integrity when fibrillin-1 is deficient. The age of each patient is indicated: (III) 48 years, (VI) 20 years (V) 20 years. Merged images on the right represent a 5.7-fold magnification of the marked area. Scale bar: 250 μm. (**B**) Co-localization of EMILIN-1 and EMILIN-3 with fibrillin-1 in dermis of MFS patient (58 years) at the DEJ. Right: 3.6-fold magnification of marked area. Scale bar: 25 μm. Arrows point to punctate signals of EMILINs colocalizing (closed arrows) and non-colocalizing (open arrows) with fibrillin-1.

We previously demonstrated that EMILINs are directly targeted to FMF, which is required to incorporate them into the murine skin ECM^28^. We therefore wanted to investigate whether structural impairment of FMF due to fibrillin-1 deficiency would affect the integrity of EMILIN networks. To address this question, we analyzed the localization and distribution of EMILINs, along with fibrillin-1, in the skin of Marfan syndrome (MFS) patients harboring various confirmed FBN1 mutations, at ages 20, 48, and 58 years (Table 1). We found that independently of the particular *FBN1* mutation the integrity and distribution of EMILIN-1, -2, and -3 was affected in all of the analyzed MFS patient skin biopsies (Fig. 6A). Particularly at the DEJ in the papillary dermis, EMILIN-1 and EMILIN-3 fibers appeared interrupted, fragmented and less organized (Fig. 6B). Elongated EMILIN-1 and fibrillin-1 positive fibers inserting perpendicularly into the DEJ were absent, instead an abundance of punctate EMILIN-1 signals was detected (Fig. 6B). EMILIN-2 and EMILIN-3 also showed a punctate pattern at the DEJ with reduced co-localization to fibrillin-1 indicating a loss of anchorage to the fibrillin microfibrillar network (Fig. 6A). These findings were independent of age as MFS patients at the ages of 20 or 58 showed the same detrimental consequences on the integrity of EMILIN-1, -2, and -3 networks (Fig. 6A,B).

**Table 1:** Specified clinical and genetic characteristics of analyzed MFS patients.

Patient	cDNA change	Mutation type	Protein change	Mutation class	Sex	Age	Biopsy location	Clinical severity	Hyper-extensible skin	Translucent skin
MFS I	c.989–1 G > C	SS	–	HI	M	58	Sternum	Moderate	n.d	n.d
MFS II	c. 4285T > A	miss	p.Cys1429Ser	DN	M	36	Sternum	Moderate	n.d	n.d
MFS III	c.989-1G > C	SS	–	HI	M	48	Sternum	Moderate	No	No
MFS IV	c.4930C > T	nons, LoF	p.Arg 1644*	HI	M	20	Sternum	Severe	No	No
MFS V	c.6616 + 1G > A	SS	-	HI	M	20	Sternum	Severe	No	No
MFS VI	c.2477G > A	miss	p.Cys816Tyr	DN	F	41	Sternum	Moderate	Yes	Yes
MFS VII	c.3932A > G	miss	p.Tyr1311Cys	DN	M	44	Sternum	Moderate	n.d	n.d

Skin biopsies and primary dermal fibroblasts were collected from MFS patients exhibiting the listed clinical and genetic characteristics.

*DN* dominant negative, *HI* haploinsuficient, LoF, *miss* missense, *nons* nonsense, *n.d.* not determined, *SS* splice site.

**Fig. 7:**
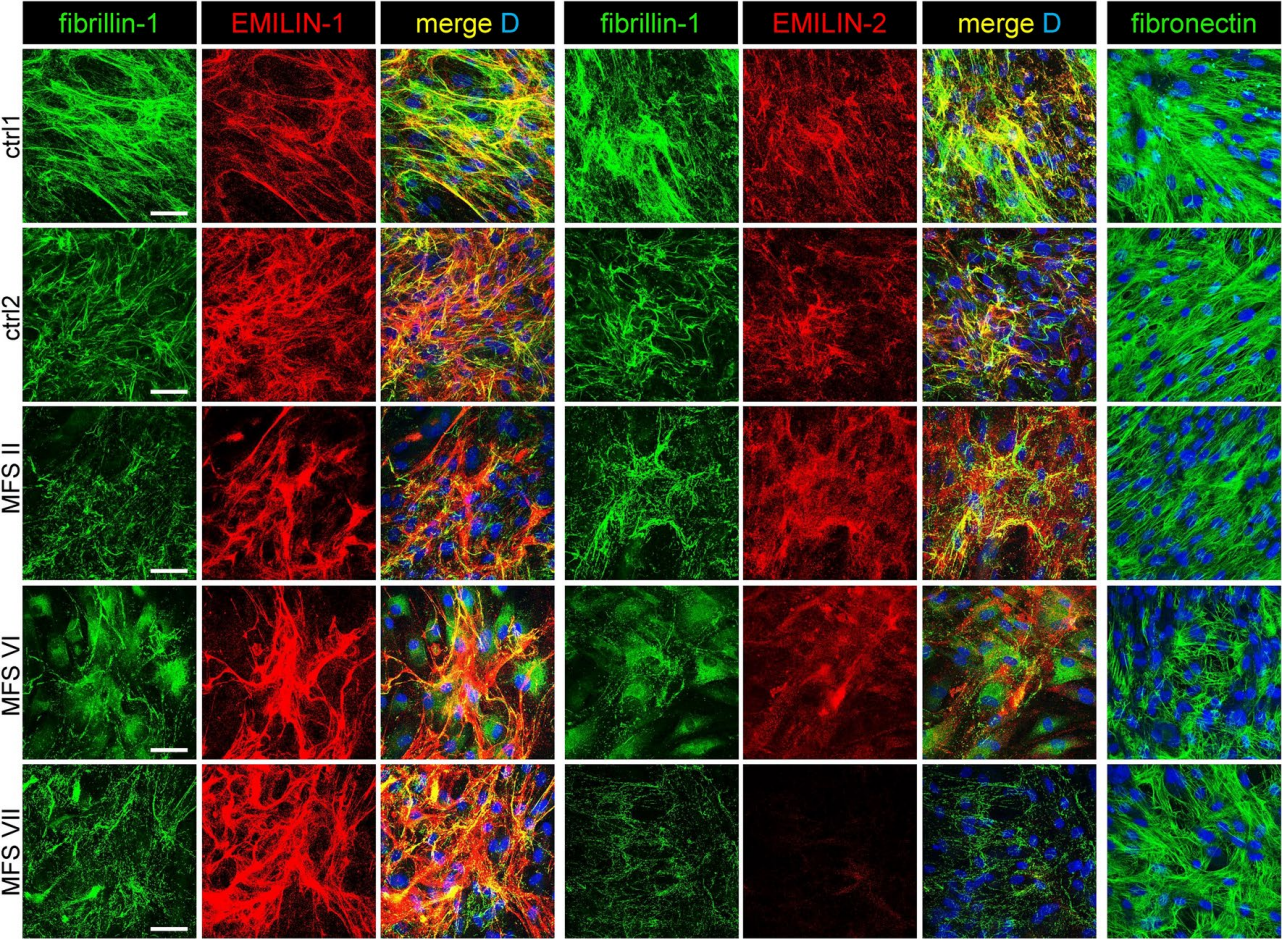
Fibrillin-1 deficiency affects EMILIN-1 and -2 fiber assembly in cultures of primary fibroblasts of MFS. ECM analysis of MFS dermal fibroblasts showed an abnormal ECM deposition of EMILINs when fibrillin-1 assembly is deficient. EMILIN-1 formed abnormally thick fibers that co-localized with the only sparsely formed fibrillin-1 fiber network. However, EMILIN-2 was only deposited as amorphous material with no evidence of fibril formation, while fibronectin assembly was not majorly affected in all analyzed cultures. Dermal fibroblasts were plated at confluence and cultured for one week followed by analysis of the ECM by confocal immunofluorescence microscopy using antibodies specific for fibrillin-1, EMILIN-1, -2, and fibronectin. Scale bar: 50 μm.

To investigate whether fibrillin-1 deficiency affects ECM assembly of EMILIN fibers, MFS patient fibroblasts were cultured and analyzed by confocal immunofluorescence microscopy. In MFS patient fibroblast cultures, fibrillin-1 staining showed a punctate pattern and a reduced number of well-organized extracellular fibers. EMILIN-1 was still incorporated into the matrix but in much thicker and strongly labeled fibrillar structures compared to control cells. In contrast, EMILIN-2 was only deposited as amorphous material with no evidence of fibril formation (Fig. 7).

### EMILIN-2 is upregulated in dermal fibrosis

**Fig. 8:**
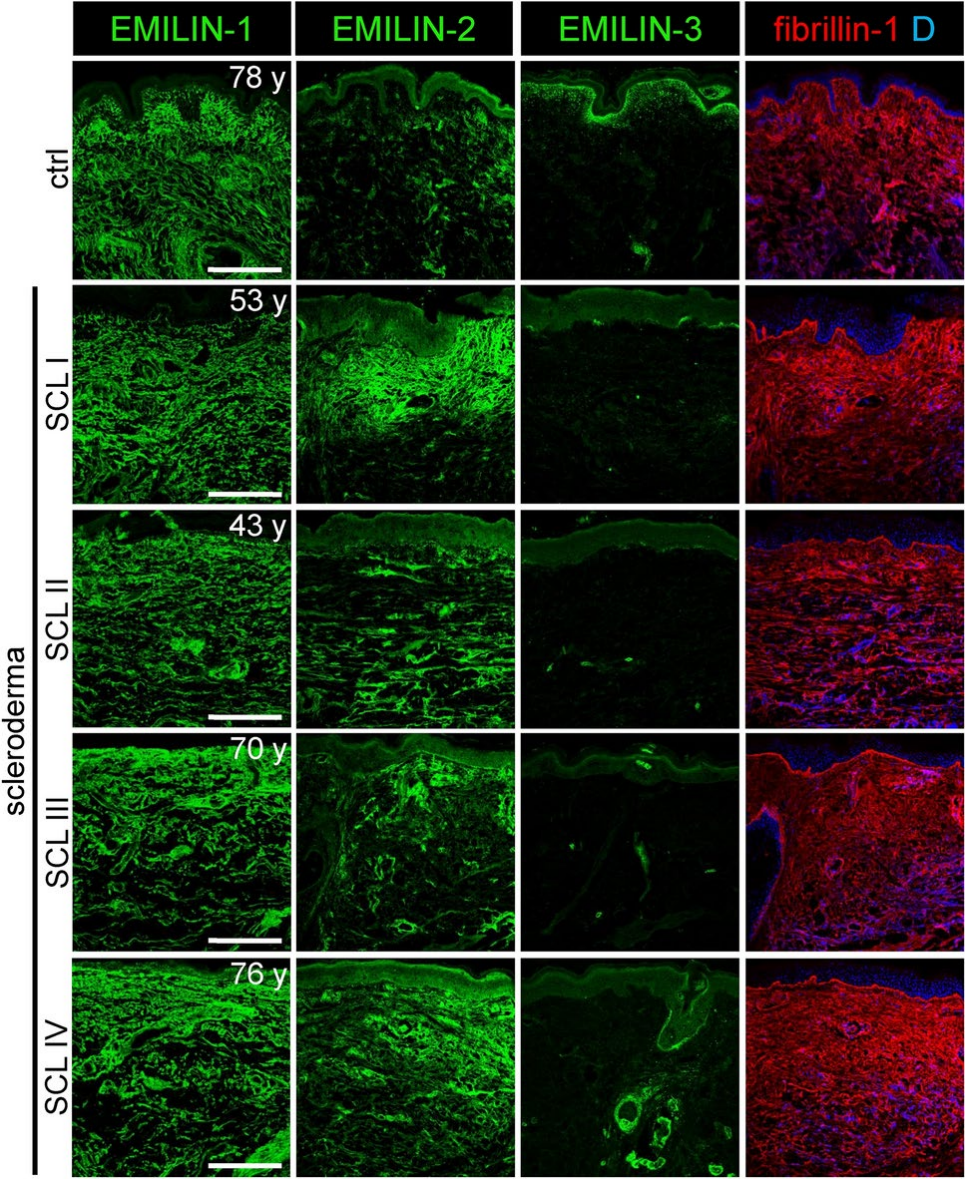
Altered localization of EMILINs within skin lesions from scleroderma patients. Confocal immunofluorescence analysis shows increased EMILIN-2 deposition in fibrotic lesions from scleroderma patients. In addition, a minor increase in EMILIN-1 signals was observed alongside a partial disruption of its typical fibrillar arrangement, whereas EMILIN-3 fibrils at the DEJ were predominantly absent in the affected areas. Skin biopsies from scleroderma patients were taken from the following anatomical locations: ctrl: anterior axillary line, SCL I: left lower arm, SCL II: right lower arm/dorsal, SCL III: left lower arm, SCL IV: left lower arm. Clinical information regarding the biopsies is summarized in supplementary Table S1. Donor age is indicated. D: DAPI. Scale bars: 250 μm.

**Fig. 9:**
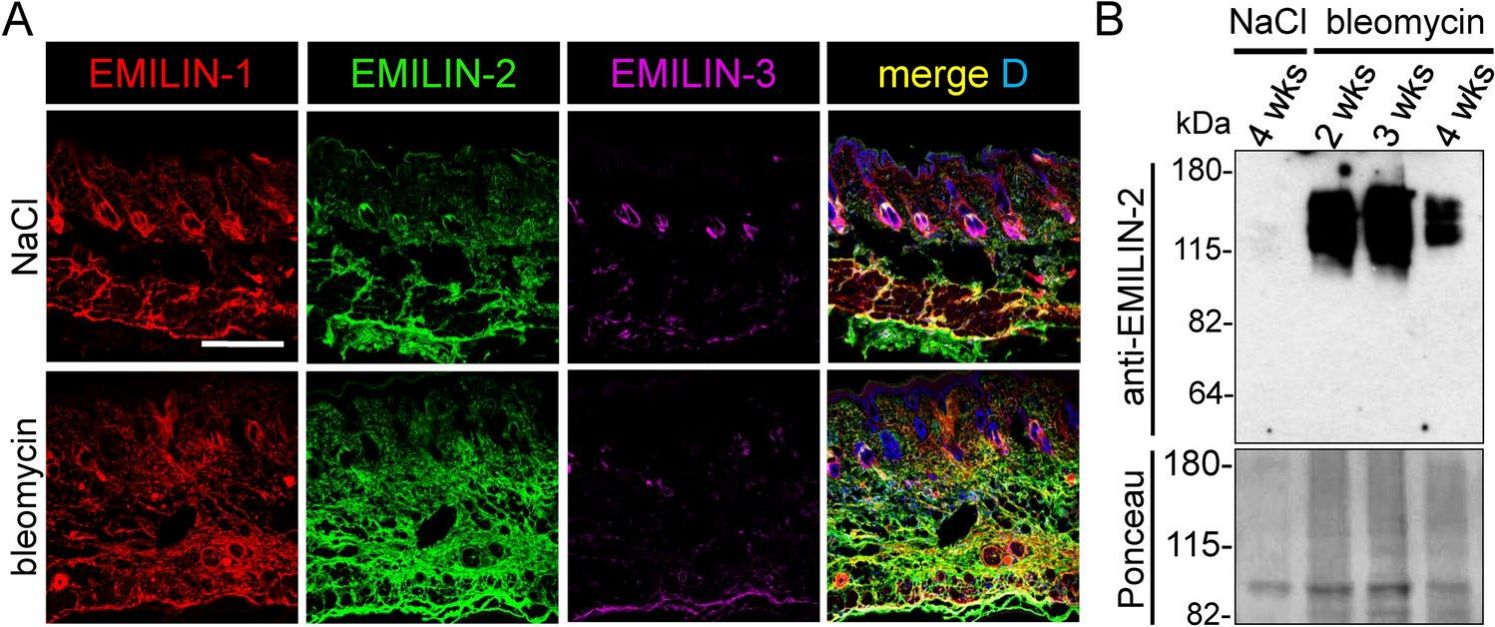
EMILIN-2 is upregulated in experimental mouse model of bleomycin-induced skin fibrosis. Increased EMILIN-2 protein deposition and synthesis upon bleomycin application in murine skin. (**A**) Deposition of EMILINs in mouse skin after 4 weeks of intradermal bleomycin (bleo) or 0.9% NaCl injection. (**B**) EMILIN-2 immunoblot analysis of skin proteins isolated during the course of bleomycin or NaCl injection. Donor age is indicated. DAPI (D) marks nuclei. Scale bar: 50 μm.

Since EMILIN-2 transcription was elevated in a murine model for scleroderma/ systemic sclerosis^35^, we investigated whether fibrotic conditions affect the ECM deposition of EMILINs. Immunofluorescence analysis revealed increased EMILIN-2 deposition in fibrotic lesions from scleroderma patients (Fig. 8, Table S2). In addition, we observed a slight increase in EMILIN-1 deposition associated with a partial loss of normal fibrillar organization, while EMILIN-3 fibrils at the DEJ were mostly lost in affected regions (Fig. 8). To investigate whether the induction of a fibrotic skin response would lead to an upregulation of EMILIN-2 expression, we analyzed skin from an experimental mouse model of bleomycin-induced skin fibrosis^36^. Immunofluorescence analysis revealed a markedly increased EMILIN-2 deposition particularly in the reticular dermis (Fig. 9A). Western blot analysis of skin extracts showed that EMILIN-2 is strongly upregulated between two and three weeks of bleomycin treatment (Fig. 9B). EMILIN-1 signals were also found to be slightly increased within the reticular dermis of bleomycin-treated skin, while EMILIN-3 signals, which only showed localization to hair-follicles, appeared to be reduced (Fig. 9A).

## Discussion

The extracellular microenvironment of the skin is vital for maintaining homeostasis by regulating biological processes such as regeneration through spatial activation of growth factors like TGF-β and Wnt^37,38^. In the hair follicle bulb and sebaceous glands, these pathways, along with Hedgehog signaling, control keratinocyte and sebocyte differentiation^39–41^. Epidermal-dermal crosstalk is facilitated by ECM components such as anchoring fibrils (collagen VII)^42^ and anchoring cords (AMACO)^43^. Understanding the distribution of key ECM components is critical for insights into skin pathology and the development of therapeutic strategies.

EMILINs are extracellular glycoproteins that regulate cell migration, differentiation, and proliferation by interacting with integrins^1,2^ and modulating TGF-β^5,6^, Wnt^10^, and Hedgehog pathways^9^. However, their role in human skin remains largely unexplored. Investigating changes in EMILIN localization during aging, photoaging, and fibrotic conditions is essential to understanding ECM-cell communication and advancing therapeutic development.

In this study, as well as in previous observations of murine skin, we noted a regular deposition of EMILIN-1 in patches onto FMF and elastic fibers within the dermis and papillary dermis (Fig. 4B), occasionally demonstrating a periodicity of approximately 100 nm^28^. FMF have a characteristic periodicity of 50–55 nm due to a staggered conformation in which about 8 monomers overlay to form the bead region^44^. The precise binding site of EMILIN-1 within the FMF assembly has yet to be mapped. However, a periodicity of about 100 nm indicates that at only every second bead EMILIN-1 signals could be detected. This suggests the existence of adaptor molecules that bind to the bead region such as microfibril associated glycoproteins (MAGPs)^45,46^ that may restrict and control EMILIN-1 deposition onto the FMF scaffold. In addition to direct targeting to FMF, interactions of EMILIN-1 with fibulins may facilitate their localization to the elastic core and direct spatio-temporal distribution within the skin. Direct binding of EMILIN-1 to fibulin-1, -2, -4, and -5 were demonstrated in interaction studies employing recombinantly expressed proteins^22,32,47^. Pull-down studies revealed direct interactions between EMILIN-2 and -3 with fibulin-1 and -2, and demonstrated an interaction of EMILIN-2 with fibulin-4^32,47^. All of these fibulins were shown to interact directly with FMF and elastic fibers^48–50^. However, additional interactions to other FMF/ elastic fiber associated ligands are possible, for instance, tissue extraction of EMILINs was only achieved under reducing conditions suggesting a covalent anchorage via disulfide bridges to unknown binding partners^11,28^.

Confocal immunofluorescence microscopy studies of EMILINs at the DEJ revealed that EMILIN-1 and EMILIN-3 co-localize with collagen VII and laminin 332 within the BM (Fig. 4). This suggests that EMILIN-1 and -3 facilitate direct contacts with basal keratinocytes. EMILIN-3 was detected to be present at the very tips of candelabra-like structures that insert perpendicular into the BM (Figs. 2, 4). A unique function of EMILIN-1 can be assumed since it forms fibrillar projections to reach beyond the BM to make contacts with basal keratinocytes^1^. Our immunogold-EM data clearly demonstrated the exact localization of EMILIN-1 directly at the plasma membrane of basal keratinocytes (Fig. 4B). As previously reported, the interaction of EMILIN-1 with the specialized integrin alpha4/alpha9 prevents hyperproliferation of basal keratinocytes^1^. This was inferred from the observation of epidermal thickening in *Emilin1*^*-/-*^. Interestingly, EMILIN-3 was found to be strongly associated with the BMs of human skin appendages as hair follicles and sebaceous glands, mirroring the distribution observed in mice^27^. Furthermore, EMILIN-3 demonstrated binding affinity to heparin^5^, indicating potential interactions with proteoglycans found in hair follicles and sebaceous glands. This suggests an involvement of EMILIN-3 in modulating the extracellular environment in skin stem cell niches by regulating the availability and distribution of various secreted factors known to be influenced by heparan sulfate proteoglycans, including Wnt, Hedgehog, and bone morphogenetic protein ligands^51^. However, it is not clear whether and how EMILIN-1 and -3 fibers are anchored to the BM and whether they assemble along with BM networks. Immunogold labeling experiments could not clarify this question, since gold particle diffusion into the lamina densa is difficult and can be best achieved by 1 nm particles^16^. The absence of detected EMILIN immunogold signals along the lamina densa does not definitively rule out the possibility of EMILIN presence in that location. Previously, it was shown that within the BM the characteristic periodicity of FMF is lost^16^. It is possible that EMILIN-1 and -3 are targeted to fibrillin-1 monomers that are arranged in sheet-forming networks that are interwoven with the nidogen-1 and -2 and laminin networks. However, functional interaction studies with recombinant proteins showed no binding of EMILINs with nidogen-1 and -2^47^. Localization of EMILIN-3 and collagen VII anchoring fibrils was observed by immunogold labeling in a neurofibroma skin biopsy (Fig. S7), which could be not observed in normal skin. This indicates that upon ECM restructuring EMILIN-3 may play a specific role and structurally support anchoring fibril integrity. In aged individuals, EMILIN-3 fibers appeared shorter, less abundant, and displayed reduced staining intensity (Fig. 5A). While aging keratinocytes showed a reduction in EMILIN-3 mRNA expression, aged papillary fibroblasts exhibited an increase in EMILIN-3 transcript levels (Supplementary Fig. S5). This suggests a potential compensatory mechanism at the transcriptional level that does not translate into detectable protein levels. Both keratinocytes and papillary fibroblasts may contribute to the production and assembly of EMILIN-3 protein, which is observed in oxytalan fibers. However, further studies are needed to clarify the cell-specific contributions to the formation of EMILIN-3 at the tips of oxytalan fibers, particularly at the dermal–epidermal junction in human skin.

Degradation and an overall reduction of EMILIN-1 and EMILIN-3 fibers were detected in aged and UV exposed skin (Fig. 5). Loss of EMILIN-3 at the tips of oxytalan fibers may reduce the pliability of skin and be indicating that epidermal-dermal connections are lost and deep wrinkles are formed. Matrix-degrading metalloproteinase (MMP) mRNAs, proteins and activities are induced in human skin in vivo within hours of exposure to UVB irradiation, and may degrade EMILIN networks in skin^52^. EMILIN-1 was reported to be degraded by MMP-3, -9 and MMP-14 as well as by neutrophil elastase in vitro^53^. Interestingly, only neutrophil elastase cleavage, an enzyme known to be upregulated and degrade ECM networks upon UV light exposure^54^, is capable to disrupt the correct folding of the EMILIN-1 gC1q which is required for multimeric assembly of EMILIN-1^55^and suppresses cellular proliferation^53^. Therefore, EMILIN-1 degradation may significantly alter the behavior of skin resident cells. Similarly, the presence of degraded EMILIN-1 fibers in the skin of MFS patients suggests that EMILIN may contribute to the pathogenesis of MFS. EMILIN-1 and -3 were shown to exert an inhibitory activity via their EMI domain on all three pro-TGF-β molecules, and thereby regulate their bioavailability^5,6^. Therefore, an impairment of EMILINs due to degradation may further contribute to dysregulated TGF-β activity caused by insufficient targeting and anchorage to FMF. A hallmark of skin features in MFS patients are striae distensae and translucent skin, both potentially caused by MMP-mediated degradation of elastic and collagen networks^56–58^. Dysregulation of FMF-targeted TGF-β and BMP growth factors can lead to elevated MMP levels, potentially resulting in the liberation of bioactive growth factors that perpetuate a vicious cycle of ECM degradation^59,60^.

In MFS fibroblast cultures, formation of thickened EMILIN-1 fibers may represent an initial rescue mechanism to correct for defective FMF assembly, while EMILIN-2 ECM incorporation failed. However, binding of EMILIN-2 to FMF could be significant in fibrotic reactions. Our data show that EMILIN-2 is upregulated in fibrotic lesions from scleroderma patients as well as upon bleomycin injection into murine skin (Figs. 8, 9). In the tight skin mouse, a genetic model of systemic sclerosis caused by a large in-frame duplication in the* Fbn1 gene*^61^ that alters FMF ultrastructure^62^, EMILIN-2 was found to be upregulated^35^. Similarly an upregulation of EMILIN-2 was found in an experimental mouse model of bleomycin-induced lung fibrosis^63^. Currently, there are no expression data available for EMILIN-2 in the lung, where only EMILIN-1 is known to be highly expressed during mouse development^64^and remains abundant in postnatal stages^22^. The observed induction of EMILIN-2 upon bleomycin treatment in both skin and lung suggests that this pathway is conserved across these tissues. This is particularly interesting given that EMILIN-2 is a known interaction partner of the Wnt-1 ligand^10^which is known to be a profibrotic cytokine in systemic sclerosis^65^. EMILIN-2 may modulate Wnt signaling in fibrotic conditions and thereby pose new therapeutic interventions. For instance, the dermal and perivascular deposition of EMILIN-2, not co-localizing with elastin (Figs. 2, 3A) suggest a role in vascular damage in fibrosis and in the regulation of vascular stability. EMILIN-3 was not detected in fibrotic lesions and may therefore be downregulated. The mechanism driving the upregulation of EMILIN-2 in fibrotic reactions remains unclear, as does the specific cell type responsible for its increased presence. It is plausible that reticular fibroblasts contribute most significantly to EMILIN-2 production, given their presence in fibrotic lesions. Transcript analysis supports this hypothesis, showing that EMILIN-2 and EMILIN-1 are highly expressed in reticular fibroblasts, while EMILIN-3 is absent (Fig. 1). Notably, EMILIN-2 expression is approximately 25% higher than EMILIN-1 in these cells (Fig. 1).

In summary, our gathered data suggest that EMILINs contribute to the specialized functional and architectural properties of cellular microenvironments in the skin. The distinct localizations and distributions of EMILINs suggest unique structural and growth factor modulating functions that vary depending on the spatio-temporal context, highlighting their non-redundant roles. Our findings suggest that EMILINs play a crucial role in skin diseases and could serve as potential diagnostic markers for conditions such as skin aging, photoaging, connective tissue diseases, and fibrotic reactions. However, further studies are needed to fully understand their functional significance in these contexts.

## Materials and methods

### Ethics statement

Clinical information on patient specimen is listed in Table 1 and Table S1. The use of human specimen involved in this study was approved by the institutional review boards at the Medical Faculty of the University of Cologne (SFB829 Ethikvotum 08-144) and Ghent University (B6702020000194)*.* Written informed consent was obtained from patients in accordance with institutional review board policies. Animal experiments were carried out in strict accordance with the German federal law on animal welfare and ARRIVE guidelines, and the protocols were approved by the “Landesamt für Natur, Umwelt und Verbraucherschutz Nordrhein-Westfalen” (permit numbers 8.87-51.05.20.13.004 and 84-02.04.2013.A069).

### Analysis of single-cell RNA sequencing data

The analyzed scRNA-seq datasets included five subjects: two younger individuals (25 and 27 years; y1 and y2) and three older individuals (53, 69, and 70 years; o1, o2, and o3). These datasets, previously described in detail^33^, are publicly available in the Gene Expression Omnibus (GEO) database under accession number GSE130973. Data analysis was performed using R (version 4.3.3) and Seurat 5.0. Quality control included filtering cells with fewer than 200 or more than 2500 features (nFeatures) and excluding cells with mitochondrial RNA content (percent.MT) exceeding 5%, to remove low-quality cells such as doublets or dead cells. Inter-individual variation and batch effects were addressed by integrating all samples using the standard integration workflow of Seurat. Pre-processing steps were initially applied to each dataset independently. UMI counts were log-normalized with a scaling factor of 10,000 using the NormalizeData function, and the 2000 most variable genes were identified with the FindVariableFeatures function. The data were scaled using ScaleData, followed by dimensionality reduction with principal component analysis (PCA) using RunPCA. Unsupervised clustering was conducted using the FindNeighbors and FindClusters functions. For the first study, the top 10 PCA dimensions were used, while 15 dimensions were utilized for the second study to construct the Shared Nearest Neighbor (SNN) graph. Clustering was performed using a modularity optimization-based algorithm at a resolution of 0.2. Visualization was achieved with the RunUMAP function, employing the first 15 PCA dimensions and default parameters. To define cell clusters, genes with enriched expression in each cluster were identified using the FindAllMarkers function. These genes were compared with known cell-type markers from the literature and the Human Protein Atlas^66^ to assign cell identities. Representative markers were used to confirm the identity of cell types typically found in human skin.

### Antibodies and reagents

A rat monoclonal antibody against mouse EMILIN-1 (clone 1007C11A8)^2^and an affinity purified rabbit polyclonal antibody (AS556) against the same antigen were kindly provided by Dr. Alfonso Colombatti (CRO-IRCCS National Cancer Institute, Aviano, Italy). Polyclonal rabbit and guinea pig anti-EMILIN-2 and anti-EMILIN-3 antisera^5,28^were kindly provided by Dr. Raimund Wagener (University of Cologne, Cologne, Germany). Anti-EMILIN-2 and anti-EMILIN-3 antibodies, all were affinity-purified, tested for cross-reactivity, and validated using knock-out tissues as previously described^27,28,67^. Affinity-purified rabbit polyclonal antibodies directed against mouse fibrillin-1 (pAb9543) and collagen VII were a kind gift from Dr. Lynn Sakai (Shriners Hospital for Children, Portland, OR, USA). Rabbit serum against laminin 332 was kindly provided by Dr. Takako Sasaki (University of Oita, Oita, Japan). Rabbit polyclonal antibody against fibronectin was from Sigma (St Louis, MO, USA), monoclonal anti-tropoelastin antibody (mab2503, clone 10B8) from Merck Millipore (Darmstadt, Germany).

### Bleomycin-induced skin fibrosis in mice

Skin fibrosis was induced in female C57BL/6N (obtained from Charles River Laboratories, Sulzfeld, Germany) mice aged 6 weeks by daily intradermal injections of bleomycin (100 µl; 1 mg/ml in 0.9% NaCl; Medac, Wedel, Germany) for 4 weeks^36^. Mice were sacrificed by cervical dislocation, only mice used for harvesting fibrotic lesions were exposed to CO_2_ (0.5 bar, 6–7 min, GasDocUnit, Medres). Skin that had been injected with 0.9% NaCl served as control. Fibrotic lesions were dissected for histological analyses, embedded in optimal cutting temperature (O.C.T.) compound (Sakura, Staufen, Germany), frozen, sectioned (8 µm) and processed for immunofluorescence staining.

### Cell culture

Primary dermal fibroblasts were isolated from human skin biopsies and cultured in Dulbecco’s Modified Eagle’s medium (DMEM GlutaMAX, Invitrogen, Carlsbad, CA) supplemented with 10% fetal bovine serum. For analysis of ECM network formation, cells were seeded on uncoated glass coverslips at 8 × 10^4^ cells/well in a 24-well plate.

### Immunoelectron microscopy

Human skin obtained from surgery was labeled using en bloc diffusion of primary antibodies^68^ followed by the appropriate secondary anti-rabbit IgG conjugated with 1-nm gold particles. The 1-nm gold was subsequently enhanced with additional gold precipitation followed by standard fixation and embedding for transmission electron microscopy.

### Immunofluorescence analysis

Human and mouse skin biopsies were embedded in Tissue-Tek (Sakura, Alphen aan den Rijn, The Netherlands), and frozen on dry ice before sectioning. Sections of 7 μm were fixed at − 20 °C in methanol/acetone, blocked in a PBS/1% bovine serum albumin solution, and subsequently incubated with primary and secondary antibodies diluted in blocking solution. Sections were mounted using Dako Fluorescence Mounting Medium (DAKO, Glostrup, Denmark). Alternatively, biopsies were fixed with 4% paraformaldehyde and processed according to standard protocols for paraffin embedding. Sections were treated with isocitrate buffer for epitope retrieval^69^ prior to incubation with the indicated antibodies. Signals were visualized with a Leica SP5 confocal laser microscope. Images were processed with ImageJ software.

### Protein extraction and immunoblotting

Skin punch biopsies (6 mm diameter) were mechanically disrupted by homogenization using a Mixer Mill (Retsch, Haan, Germany) for 2 min at 30 Hz in RIPA buffer (150 mM NaCl, 50 mM Tris base, 0.1% sodium dodecyl sulfate, 12 mM Na-deoxycholate, 1% Nonidet P-40, pH 8), supplemented with protease inhibitor cocktail (Sigma-Aldrich). Debris was removed by centrifugation. Protein concentration was determined by BCA assay (Pierce). Equal volumes of extracts were loaded.

## Supplementary Information

Supplementary Video 1.

Supplementary Information 1.

Supplementary Information 2.

## Data Availability

All data generated or analyzed during this study are included in this published article and its supplementary information files. The scRNA-seq datasets analyzed are publicly available in the Gene Expression Omnibus (GEO) database under accession number GSE130973. The R code used for data analysis is available from the corresponding author upon request.
