# Supplementary material for: New insights into the structural role of EMILINs within the human skin microenvironment
Source: Sci Rep. 2024 Dec 5;14:30345. doi: 10.1038/s41598-024-81509-5 (PMC11621341; doi:10.1038/s41598-024-81509-5)
Supplement: Supplementary file 2 — Supplementary Information 1. [file 41598_2024_81509_MOESM2_ESM.pdf]

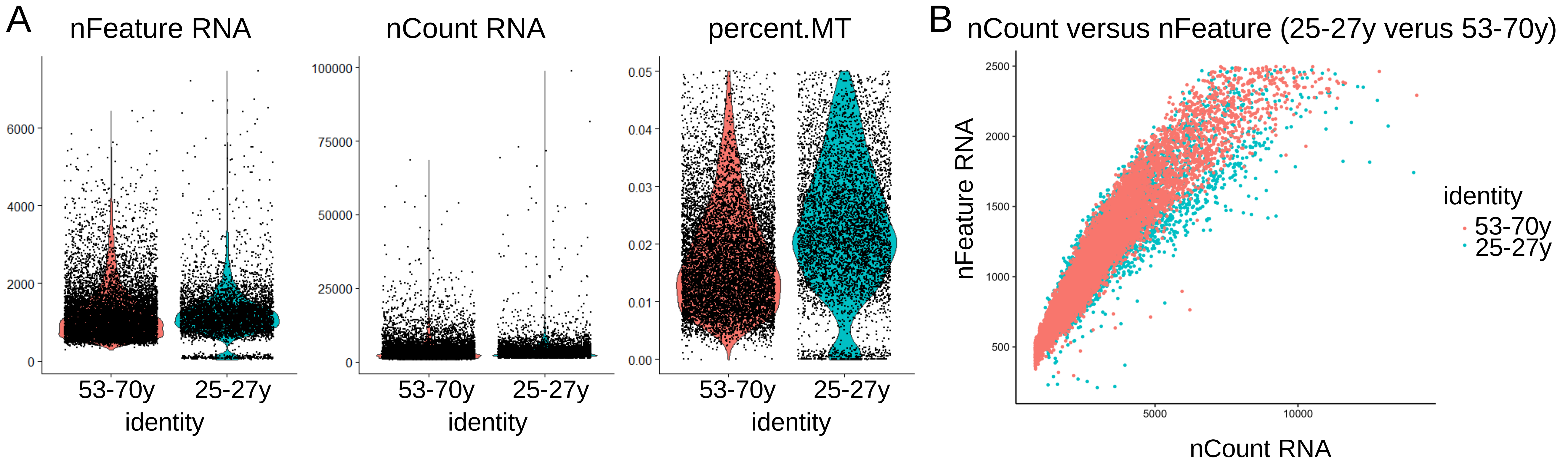


**Supplementary Figure S1: Quality control metrics for single-cell RNA sequencing data from human skin samples (25-27 and 53-70 years of age).** (A) Violin plots representing quality control metrics across all cells, including the number of unique genes detected per cell (nFeature), the total number of RNA transcripts detected per cell (nCount), and the percentage of mitochondrial RNA (percent.MT). Cells were filtered to retain those with 200–2,500 detected features and a mitochondrial RNA content below 5% to exclude potential low-quality cells and doublets. (B) Scatter plot showing the relationship between nFeature and nCount, providing insight into cellular diversity and aiding in the identification of potential outliers or low-quality cells.

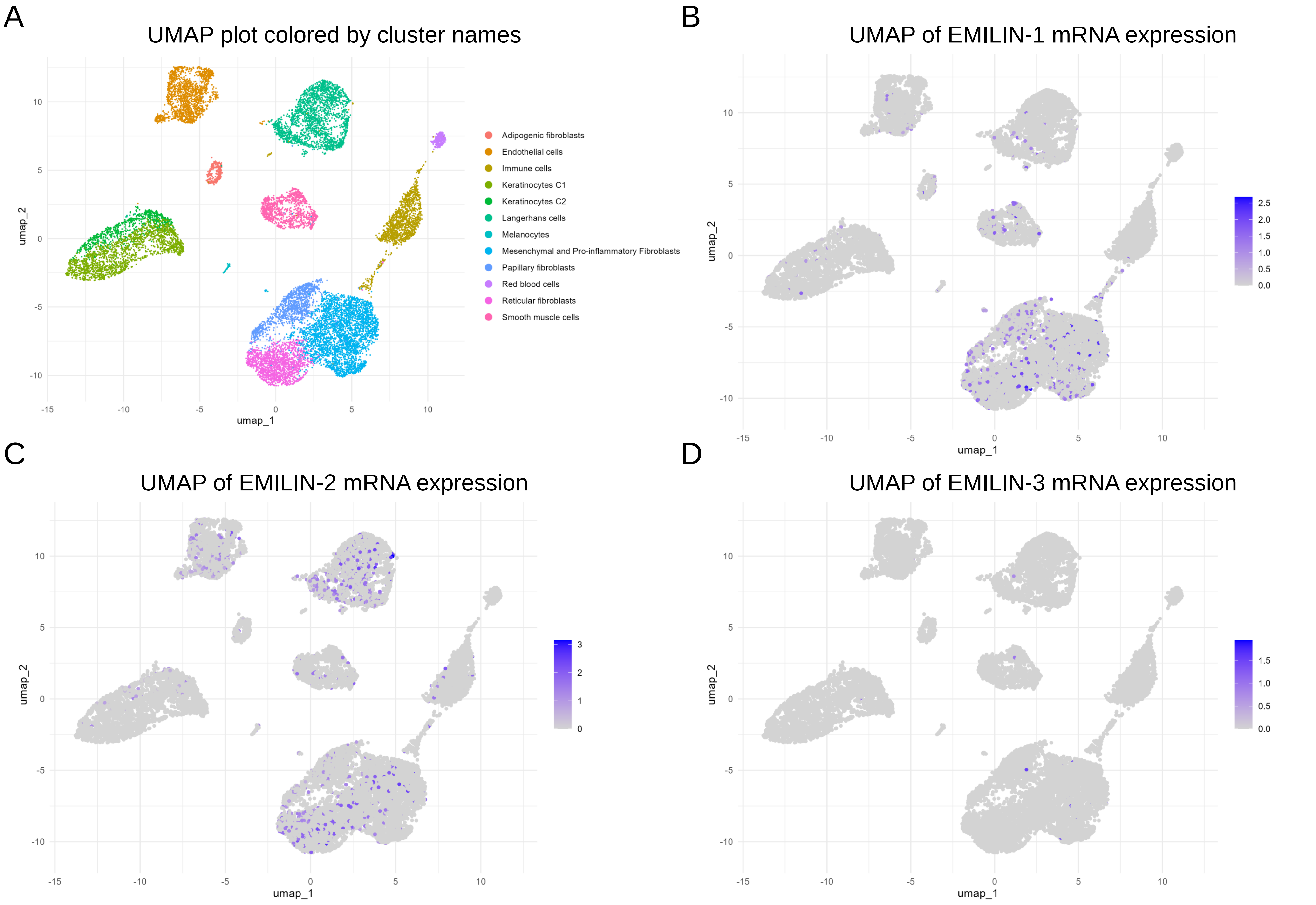


**Supplementary Figure S2: UMAP visualization of single-cell RNA-sequencing data from human skin samples.** (**A**) 12 distinct cell clusters, each representing a transcriptionally unique cell type or state, including keratinocytes, fibroblasts, immune cells, endothelial cells, and melanocytes. Data were collected from five healthy subjects (two at 25 and 27 years of age and three at 53, 69, and 70 years of age). (**B**) Expression of EMILIN-1 transcript levels across different clusters. (**C**) Expression of EMILIN-2 transcript levels across different clusters. (**D**) Expression of EMILIN-3 transcript levels across different clusters. In panels B–D, the blue gradient represents expression levels, with darker shades indicating higher expression.

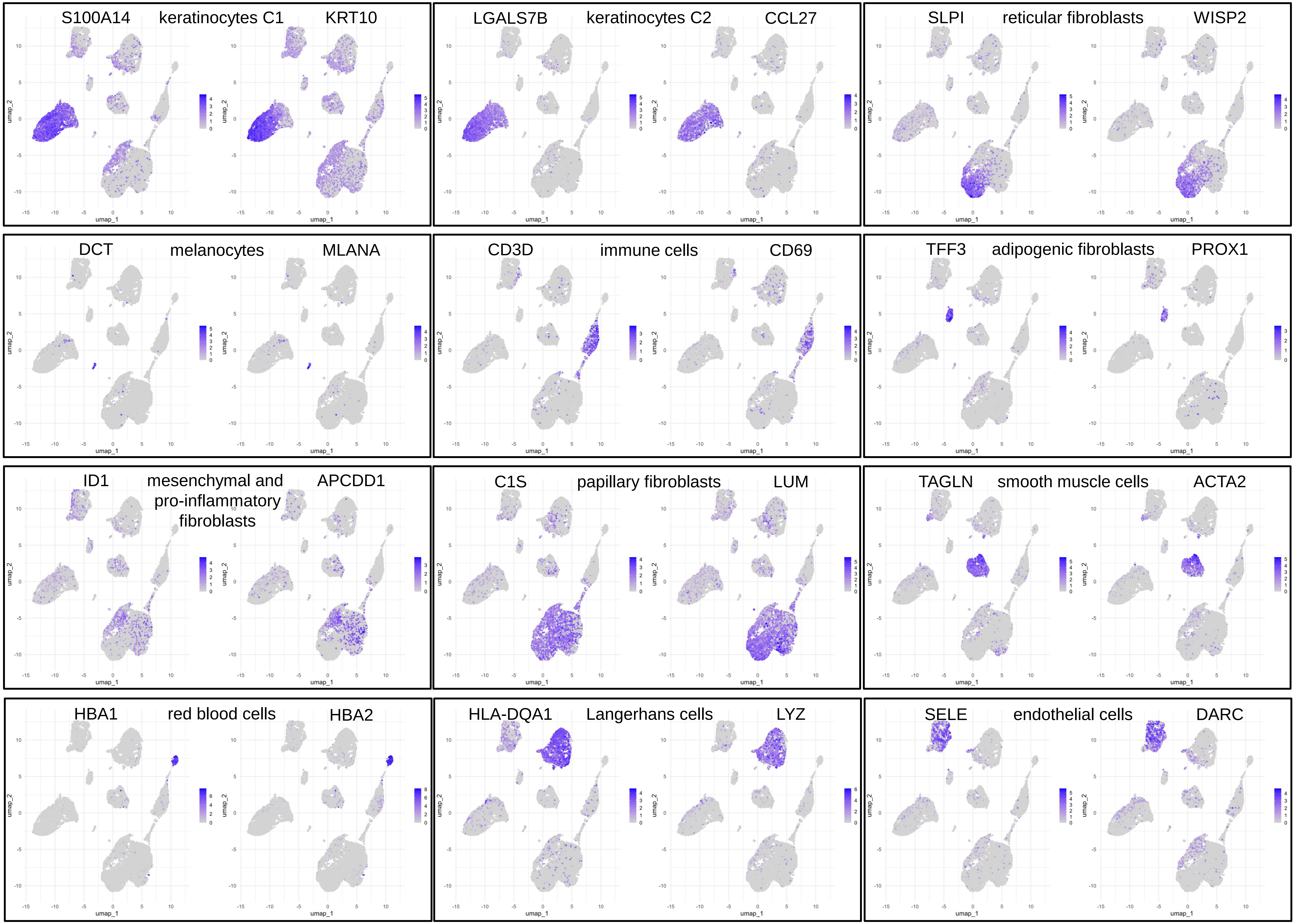


**Supplementary Figure S3: UMAP plots showing marker gene expression across 12 identified cell type clusters in human skin.** For each cell type cluster, UMAP plots of two specific marker genes are shown, highlighting distinct cellular populations. The scRNA-seq datasets were obtained from sun-protected skin areas of five healthy male Caucasian donors (two at 25 and 27 years of age and three at 53, 69 and 70 years of age). The analyzed data are available from the Gene Expression Omnibus (GEO) database (accession number GSE130973).

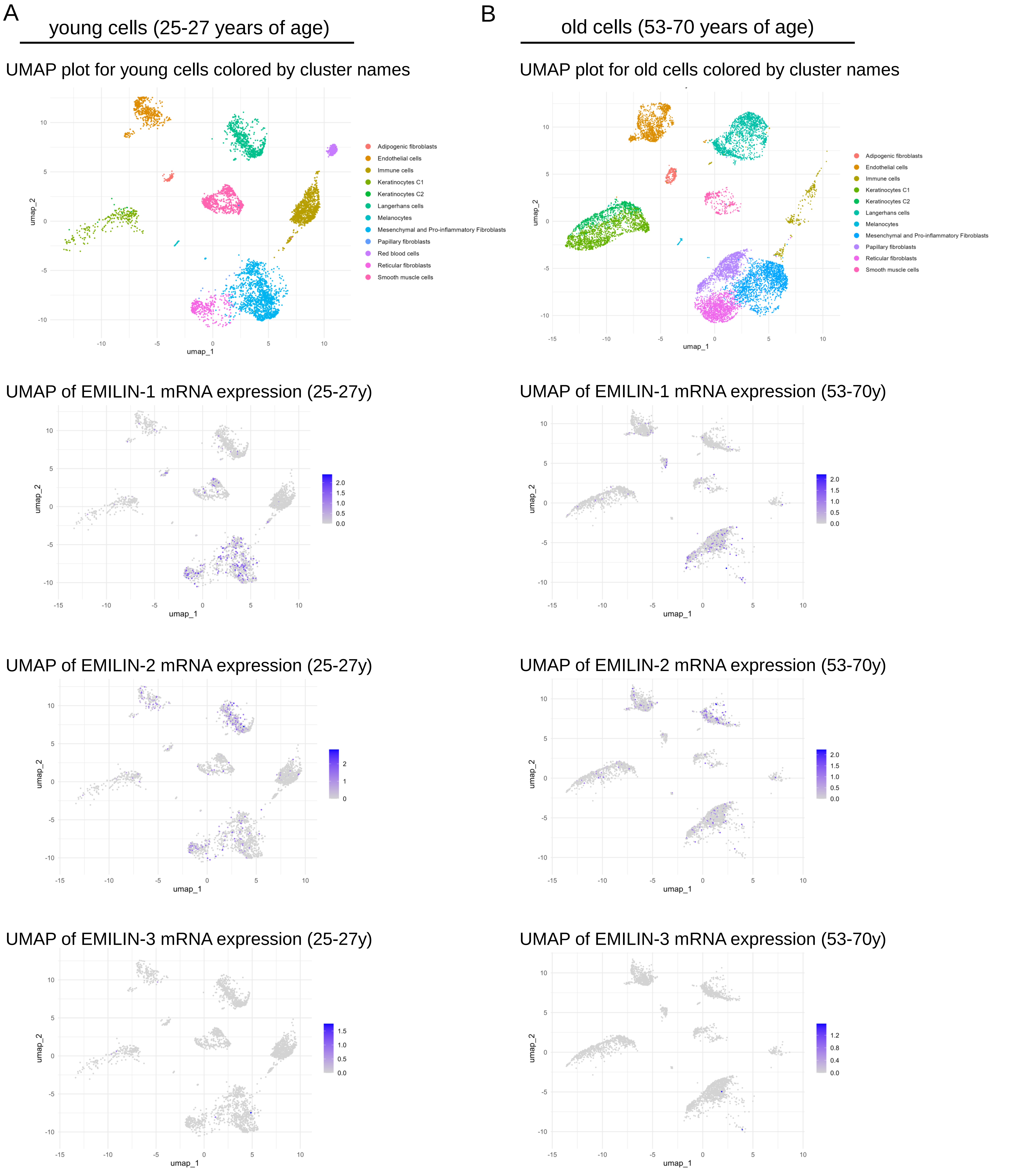


**Supplementary Figure S4: UMAP visualization of 12 distinct cell clusters in human skin, separated by age.** (A) UMAP plots for cells from two healthy donors at the ages of 25 and 27 years are color-coded by cluster names in the top panel. The lower panels depict the expression levels of EMILIN-1, -2, and -3, where the blue gradient indicates expression levels, with darker shades representing higher expression. (B) UMAP plots for cells from three healthy donors at the ages of 53, 69 and 70 years follow the same layout, with the top panel color-coded by cluster names and the lower panels showing EMILIN-1, -2, and -3 expression levels.

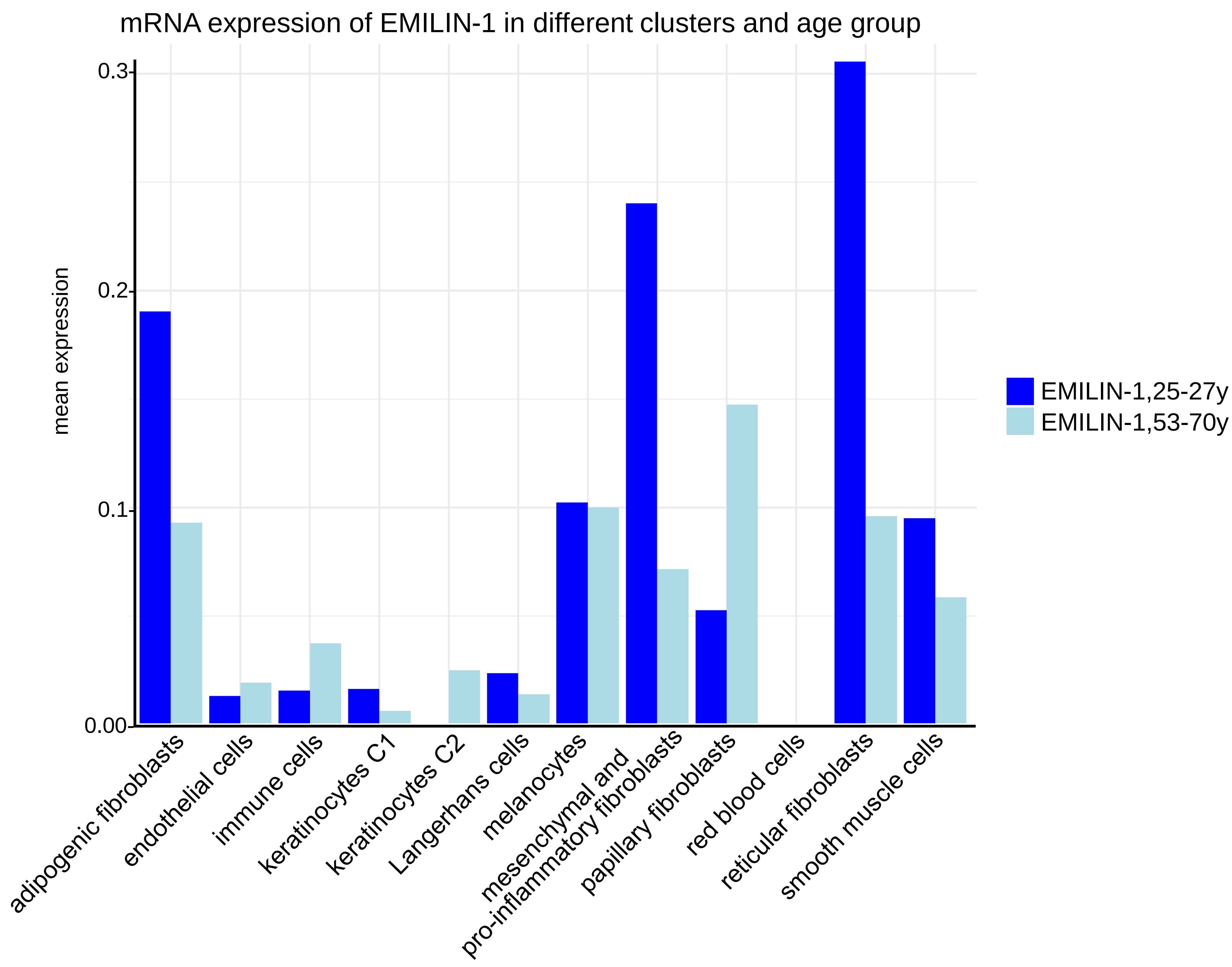


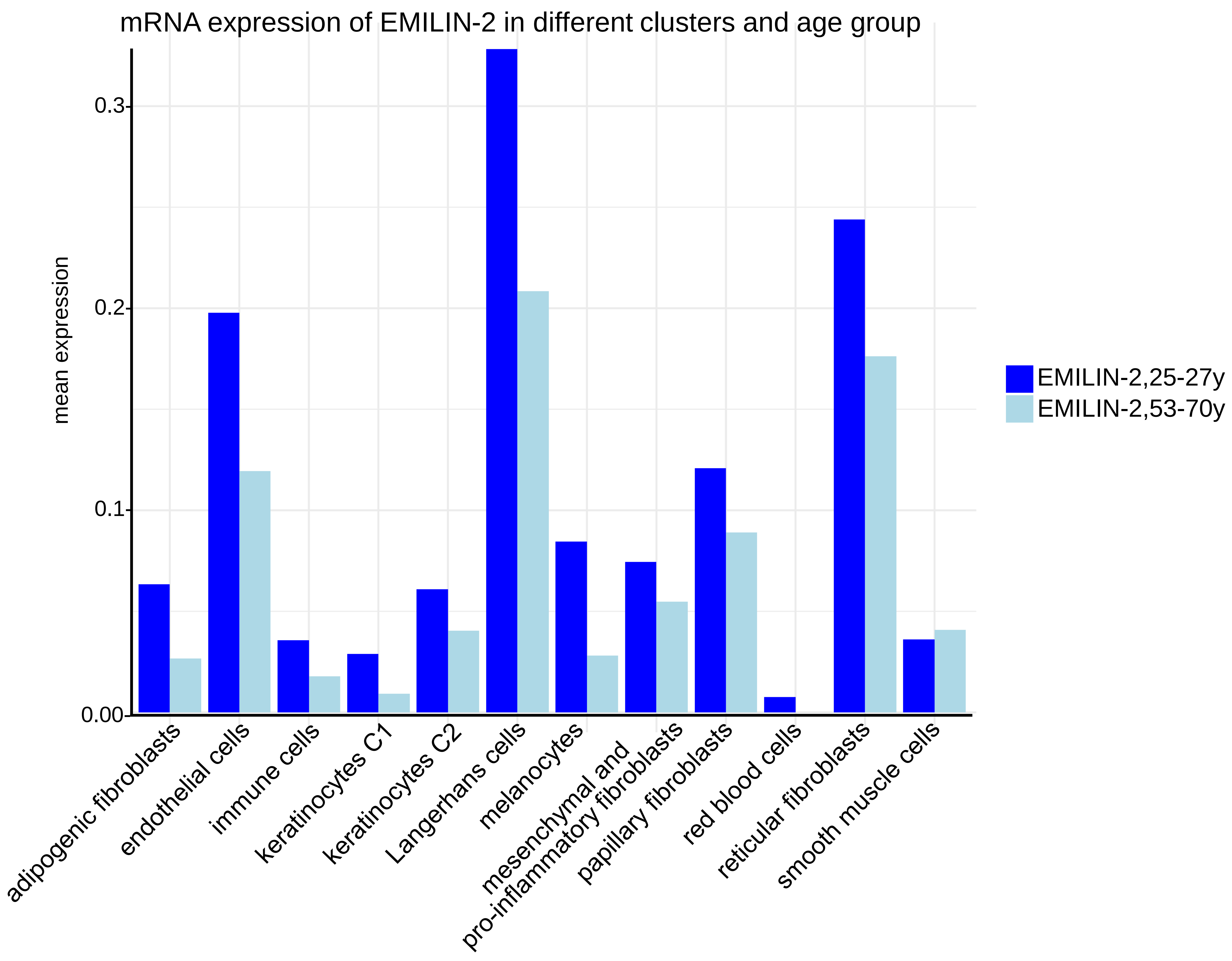


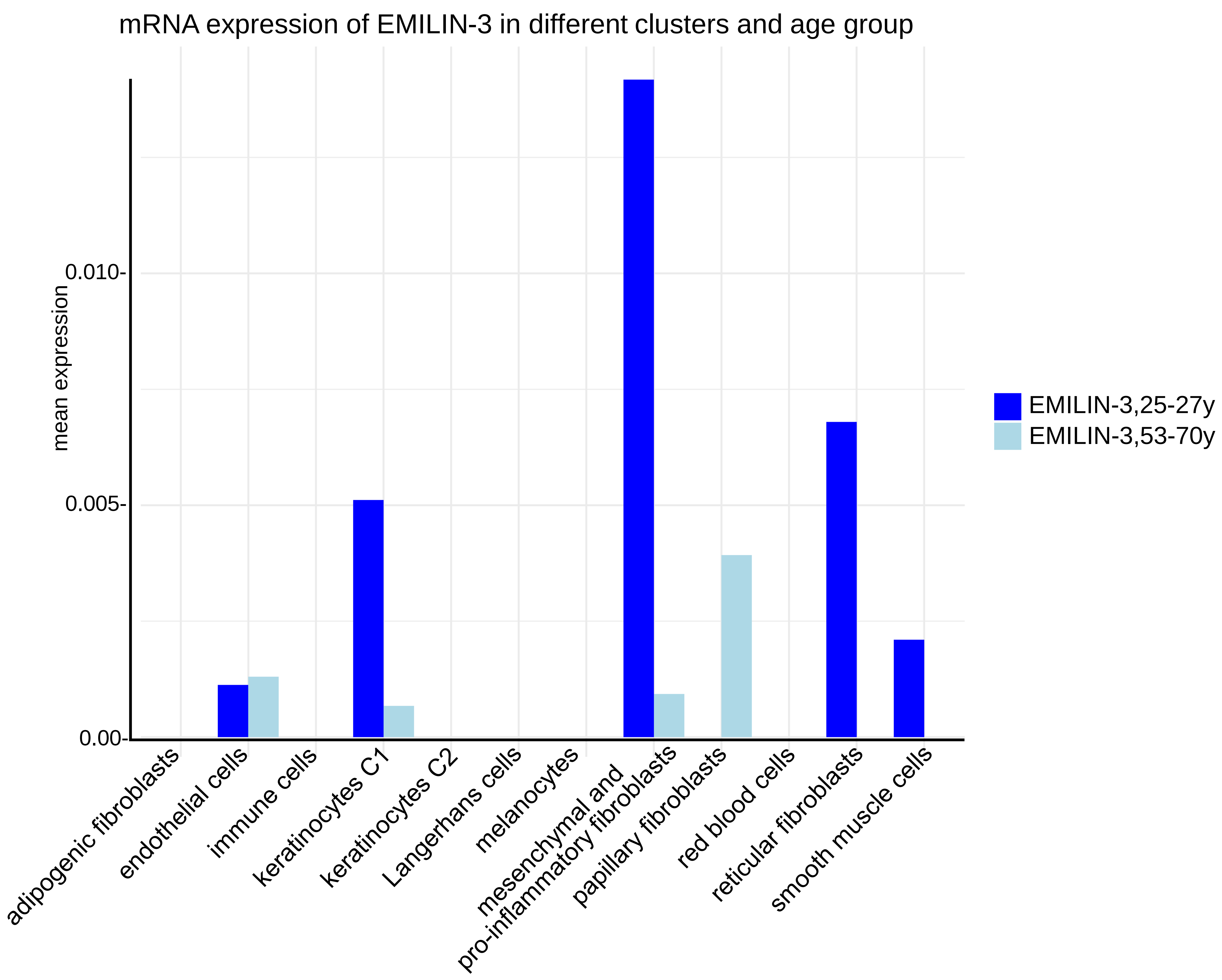


**Supplementary Figure S5: Mean expression levels of EMILIN-1, -2, and -3 across 12 identified skin cell clusters in the analyzed dataset.** EMILIN expression levels in human skin were compared between younger subjects aged 25–27 years (blue bars) and older subjects aged 53–70 years (sky-blue bars). Cell clusters were characterized based on the expression of specific marker genes (Supplementary Fig. S3, Supplementary Table S1). Mean expression levels were calculated as the average number of reads per cell for each *EMILIN* gene across clusters.

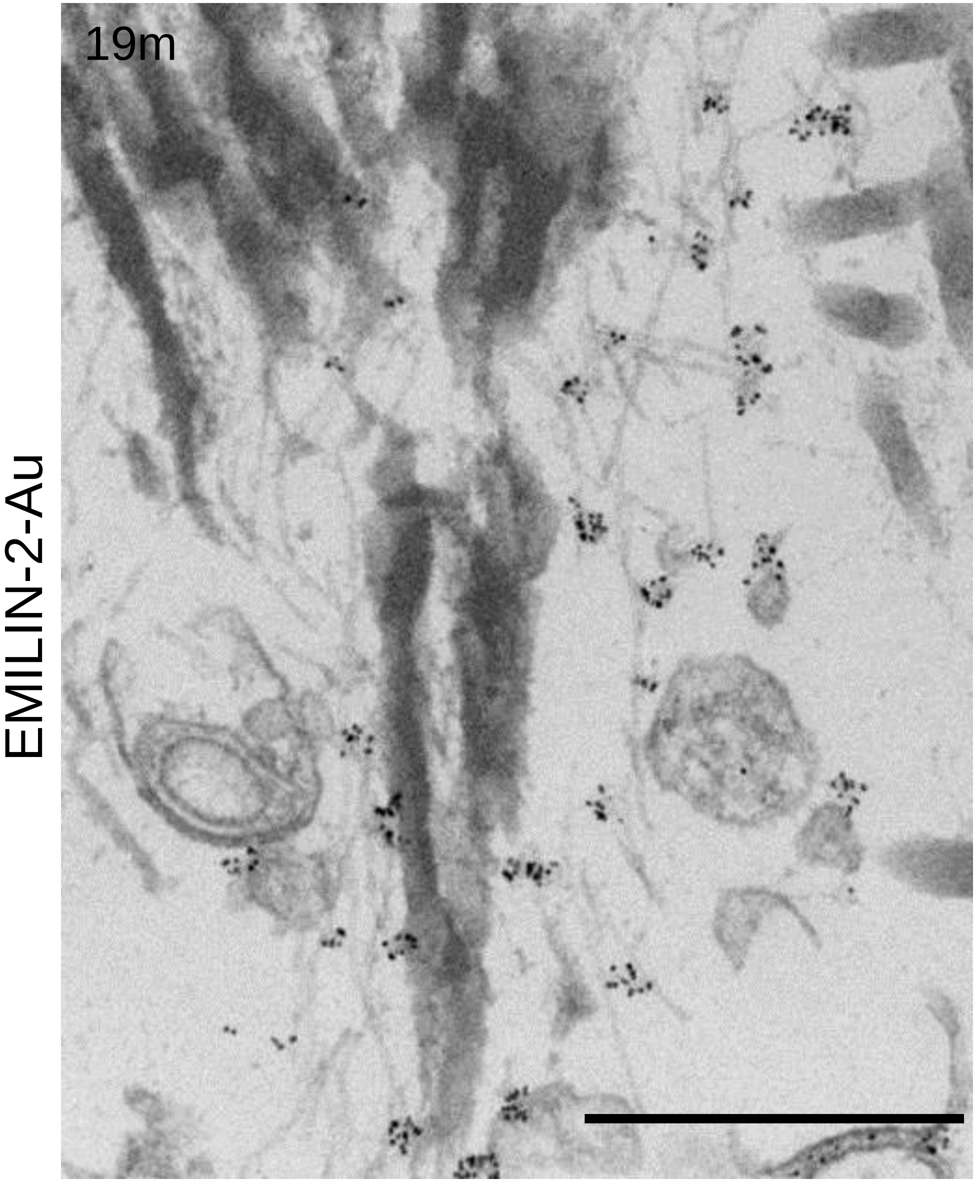


**Supplementary Figure S6. Immunogold-EM of Emilin-2 in 19-month old human dermis.** 1 nm-gold labeled anti-EMILIN-2 antibodies show localization to fibrillin microfibrils associated with elastin. Scare bar 500 nm.

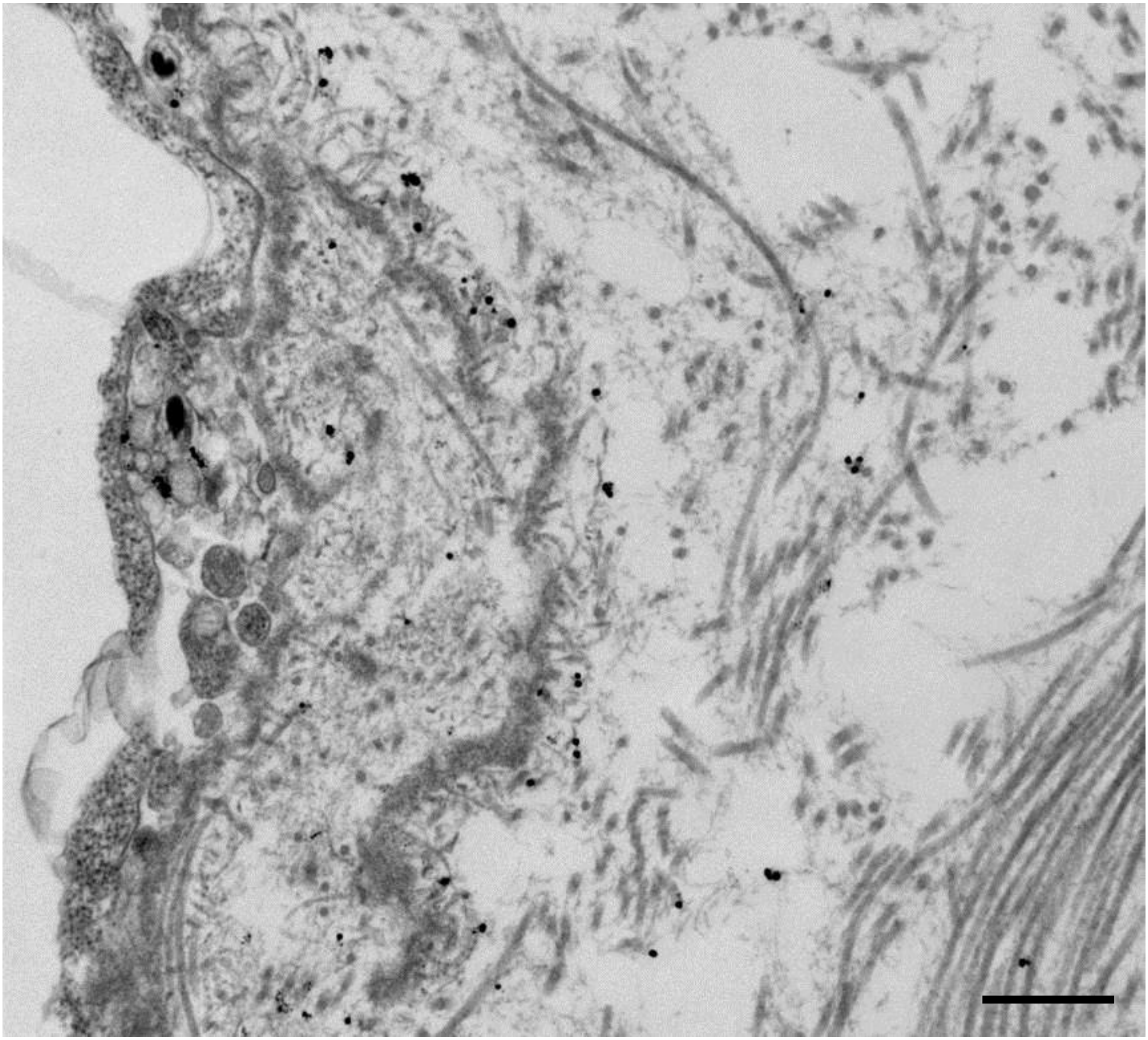

**Supplementary Figure S7. Localization of EMILIN-3 investigated by immunogold-EM in 23 year-old human neurofibroma skin.** Gold-labeled EMILIN-3 antibodies localized to anchoring fibrils, especially near basement membrane duplication. Scale bar: 500 nm.

**Supplementary table S2: Clinical information of systemic sclerosis patient biopsies.**

| patient | gender | age (years) | localization of biopsy site | diagnosis |
|---|---|---|---|---|
| SCL I | female | 53 | left lower arm / distinctly sclerotic | systemic scleroderma |
| SCL II | female | 43 | right lower arm, dorsal | systemic scleroderma |
| SCL III | female | 70 | left lower arm | systemic scleroderma |
| SCL IV | female | 76 | left lower arm | systemic scleroderma |

# Original western blots

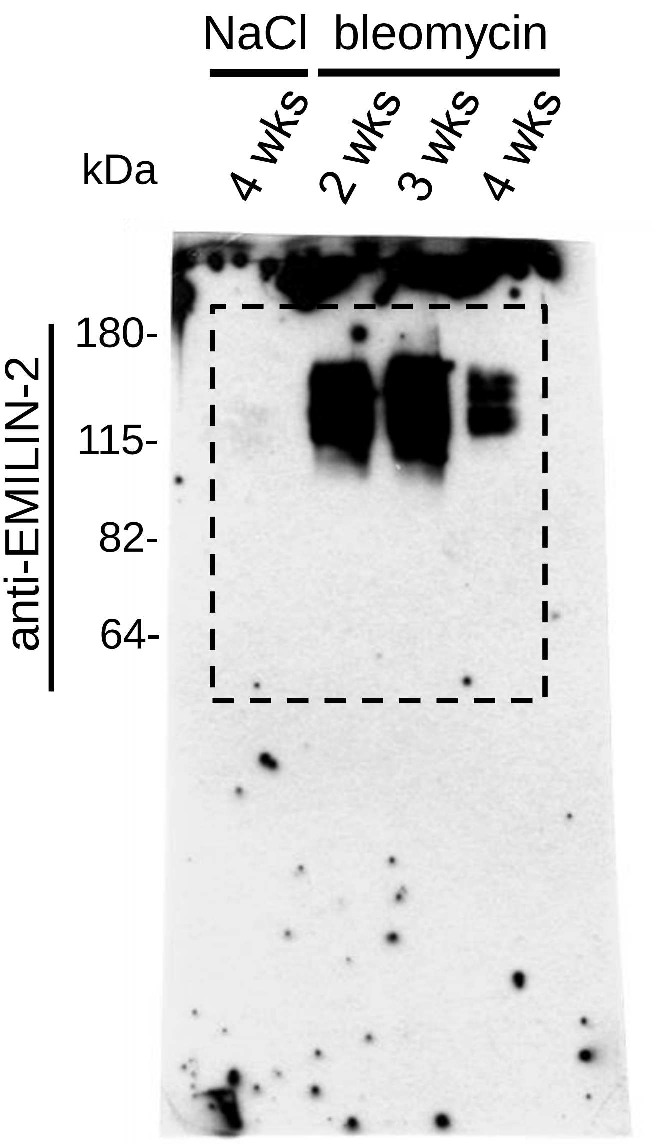


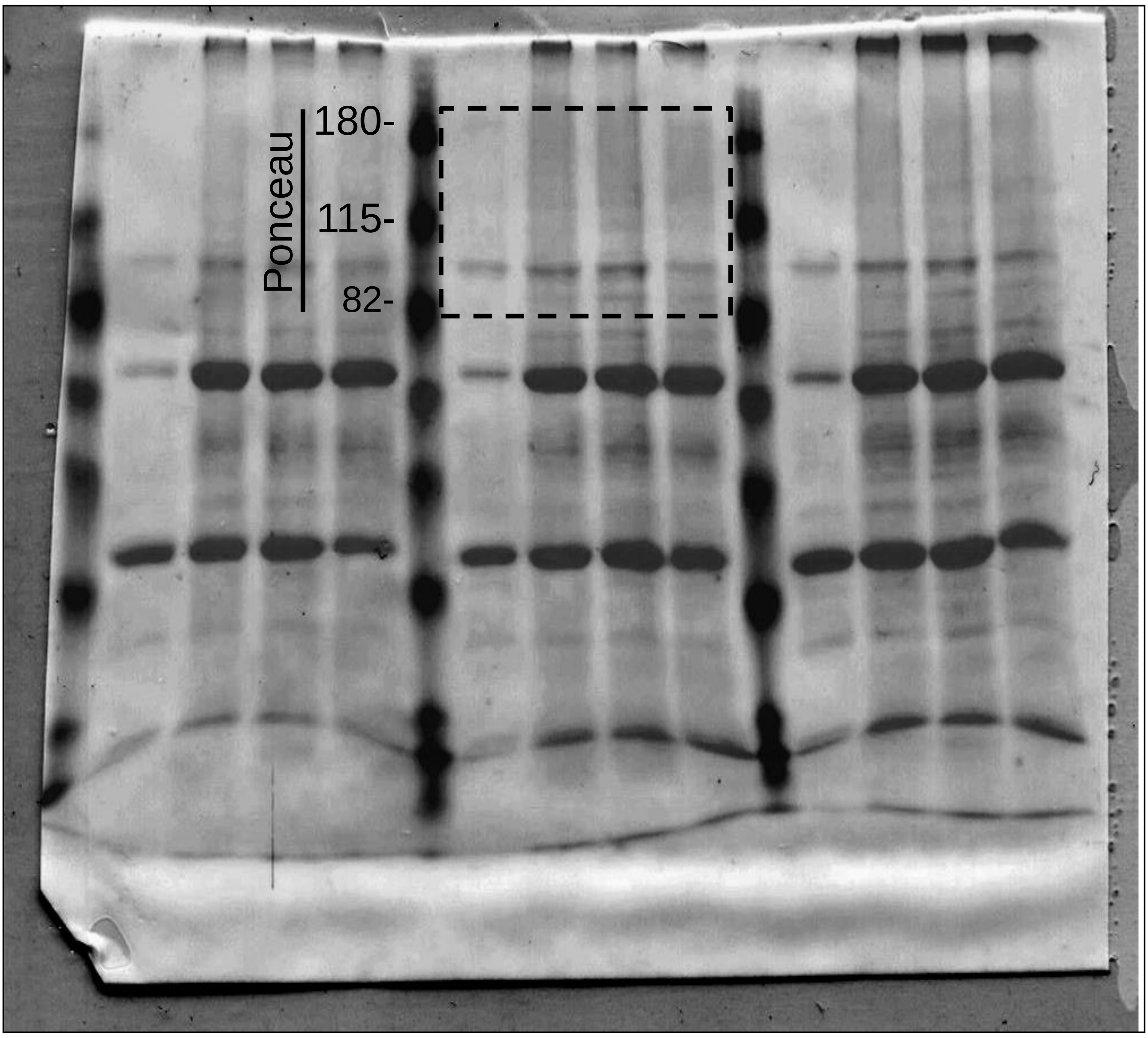


Figure 9B
